# Ozone as a Next-Generation Sterilization Process in Tissue Engineering and Regenerative Medicine: Critically Bridging Product Stability, Safety and Clinical Translation

**DOI:** 10.3390/molecules31122045

**Published:** 2026-06-11

**Authors:** Chris Manglaris, Niki Karipidou, Eleni Manolakaki, Eirini Koummati, Theodora Choli-Papadopoulou, Petros T. Koidis, Amalia Aggeli, Nikolaos Michailidis

**Affiliations:** 1 Department of Chemical Engineering, Faculty of Engineering, Aristotle University of Thessaloniki, University Campus, 54124 Thessaloniki, Greece; cmanglaa@cheng.auth.gr (C.M.); karipidn@cheng.auth.gr (N.K.); emanolaka@ece.auth.gr (E.M.); ekoummati@gmail.com (E.K.); 2 School of Chemistry, Aristotle University of Thessaloniki, University Campus, 54124 Thessaloniki, Greece; tcholi@chem.auth.gr; 3 School of Dentistry, Faculty of Health Sciences, Aristotle University of Thessaloniki, University Campus, 54124 Thessaloniki, Greece; pkoidis@dent.auth.gr; 4 Department of Mechanical Engineering, Faculty of Engineering, Aristotle University of Thessaloniki, University Campus, 54124 Thessaloniki, Greece

**Keywords:** ozone sterilization, TERM applications, scaffold integrity, antimicrobial activity, bioactivity

## Abstract

Tissue engineering and regenerative medicine (TERM) rely on advanced biomaterials and scaffolds that require strict sterilization without sacrificing their structural and functional properties. Conventional sterilization methods, including steam, ethylene oxide, and gamma irradiation, often compromise scaffold integrity, alter surface chemistry and/or leave toxic residues. Ozone (O_3_) has emerged as a promising alternative sterilant because of its strong oxidizing potential, broad-spectrum antimicrobial activity, and residue-free decomposition. Importantly, ozone sterilization can preserve—and in some cases enhance—scaffold bioactivity by maintaining cytocompatibility and favorable surface chemistries that support cell adhesion and differentiation. This review critically evaluates the role of ozone sterilization in the context of TERM applications, focusing on its physicochemical properties, disinfection kinetics, material compatibility and regulatory perspectives. Evidence from studies on polymethyl methacrylate (PMMA) scaffolds, bone implants, and hydrogel-based systems suggests that, under optimized conditions, ozone can achieve high sterilization efficacy without significant degradation of mechanical or chemical properties. However, challenges related to process validation, health and safety considerations, and scalability remain. The review highlights opportunities for integrating ozone into automated biomanufacturing workflows and identifies key research gaps to support the broader adoption of ozone sterilization in TERM applications.

## 1. Introduction

Sterility is a fundamental prerequisite for all materials used in tissue engineering and regenerative medicine (TERM), including polymer scaffolds, hydrogels, bioinks, and implantable devices, as inadequate sterilization poses significant risks of post-implantation infection. Conventional sterilization methods, such as steam autoclaving, ethylene oxide (EtO), gamma irradiation, and hydrogen peroxide plasma, remain widely used but often impose high thermal or oxidative loads, may leave toxic residues, or induce structural and chemical degradation, particularly in sensitive biomaterials [1]. Hydrogels, which possess high water content and intrinsic structural fragility, exemplify materials highly vulnerable to these conventional approaches, prompting a search for gentler yet equally effective alternatives. Emerging sterilization technologies such as ozone, supercritical CO_2_, non-thermal plasma, and ultraviolet-C (UV-C) irradiation have therefore gained attention as potential next-generation strategies capable of overcoming long-standing challenges in preserving biomaterial integrity while achieving robust sterility.

Among these, ozone sterilization is particularly promising due to its strong bactericidal and virucidal activity, low operating temperatures, and residue-free mechanism of action [2]. Although ozone is well established in water and air treatment, its application to TERM materials remains relatively novel and insufficiently explored. This gap is especially relevant as the field increasingly relies on advanced biomaterials and complex constructs whose safety and clinical translation depend on sterilization strategies that do not compromise physicochemical, mechanical, or biological performance. The growing complexity of TERM devices—from conventional scaffolds to cell-laden hydrogels and 3D-printed constructs—demands sterilization methods that preserve biocompatibility, maintain structural fidelity, and comply with regulatory standards governing medical device safety [3,4,5,6,7].

Sterilization must not only eliminate microorganisms, including vegetative cells, spores, fungi, and viruses, but also ensure that the material’s functional properties remain unaltered [8]. This challenge is amplified in 3D bioprinting, where bioinks composed of natural polymers or biological macromolecules exhibit high susceptibility to microbial contamination and are sensitive to temperature and oxidative stress. Microbial contamination can jeopardize cell viability in vitro and, upon implantation, may lead to severe inflammatory responses, abscess formation, or graft failure [9]. Accordingly, sterilization must be applied either to entire bioprinted structures or to individual components prior to bioink preparation, requiring strictly controlled aseptic workflow conditions.

Selecting an appropriate sterilization method therefore involves balancing sterility assurance, material compatibility, cost-effectiveness, and validation feasibility. While well-established sterilization methods are effective for traditional medical materials, their application to modern TERM constructs—including biologics, hybrid systems, and combination devices—is not straightforward [10]. Material cleanliness, contamination level, device geometry, porosity, and chemical reactivity all influence sterilization outcomes and must be considered when designing a suitable protocol. Importantly, no single sterilization process is universally compatible with the diverse range of biomaterials used in TERM, and techniques that rely on heat, moisture, or high-energy irradiation may cause irreversible damage to polymers and other sensitive substrates [11].

Sterilization can unintentionally alter surface characteristics, crosslinking density, molecular weight distribution, or rheological properties—features that are critical for scaffold performance and for bioink printability. In 3D bioprinting workflows, sterilization-induced changes may necessitate extensive re-optimization of printing parameters, as most optimization is traditionally conducted under non-sterile conditions [9]. These limitations highlight the urgent need for alternative sterilization strategies that are both effective and compatible with the delicate and often multifunctional materials used in modern TERM applications.

**Figure 1:**
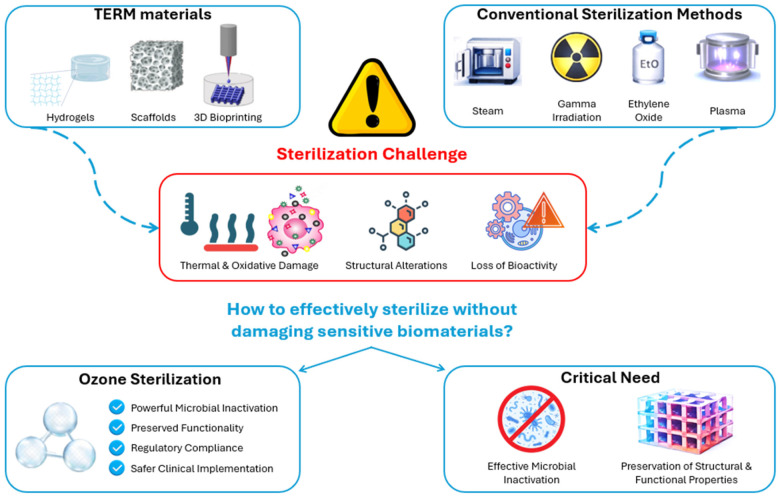
Conceptual overview of the sterilization challenge in Tissue Engineering and Regenerative Medicine (TERM): Positioning Ozone as a Next-Generation Sterilization Process.

In view of these challenges, as summarized in Figure 1, this review critically examines ozone as a next-generation sterilization method for TERM, evaluating its mechanisms of action, compatibility with advanced biomaterials, impact on structural and functional integrity, regulatory considerations, and potential pathways for clinical translation. The review was developed using a structured literature screening and thematic integration approach. Given the multidisciplinary nature of the field, the literature spanning biomaterials science, sterilization engineering, microbiology, polymer chemistry, and regulatory science was integrated to provide a comprehensive and translational perspective. Relevant studies were identified primarily through major scientific databases and publisher platforms, including Scopus, Web of Science, PubMed, Google Scholar, ScienceDirect, SpringerLink, Wiley Online Library, MDPI, ACS Publications, and Taylor & Francis Online, with additional references from engineering- and microbiology-focused sources where relevant. Particular emphasis was placed on studies published between 2020 and 2025 addressing advanced TERM constructs such as hydrogels, electrospun scaffolds, decellularized matrices, bioinks, and 3D-printed biomaterials, while foundational earlier studies were retained for mechanistic and historical context. Literature selection prioritized studies investigating ozone sterilization mechanisms, process parameters, microbial inactivation, biomaterial compatibility, validation considerations, and comparisons with established or emerging low-temperature sterilization technologies.

By consolidating current evidence and identifying existing gaps, this critical review aims to position ozone within the broader landscape of emerging sterilization technologies and assess its potential to bridge the long-standing divide between robust sterilization, material stability, and successful clinical implementation. Beyond conventional sterilization paradigms, interdisciplinary advances spanning chemical engineering, materials science, and microbial biotechnology continue to refine ozone-based systems, supporting their transition from environmental applications to biomedical sterilization platforms [12,13].

The specific contribution of this review is therefore not simply to list ozone among alternative sterilization techniques, but to define the process engineering and biomaterial compatibility questions that currently limit its use in TERM. Existing reviews have addressed either general scaffold sterilization, hydrogel sterilization, or medical-device decontamination; however, they rarely integrate ozone-specific dose delivery, humidity dependence, spore validation, porous-scaffold penetration, material-derived oxidation products, and TERM-specific biological performance in a single translational framework [1,3,6,14,15,16]. The unique knowledge gap addressed here is the absence of material-specific ozone sterilization windows that link microbial lethality with preservation of scaffold architecture, surface bioactivity, extractables/leachables safety, and regulatory validation. This review therefore aims to bridge the gap between ozone as a broad antimicrobial technology and ozone as a reproducible sterilization process for advanced regenerative medicine constructs.

## 2. Traditional Sterilization Methods

Sterilization is an essential prerequisite for clinical translation, yet biomaterials used in tissue engineering and regenerative medicine (TERM)—particularly biodegradable and bio-responsive constructs—are far more sensitive to sterilization than inert, non-degradable polymers. Conventional methods can profoundly alter critical physicochemical and biological properties, including molecular weight, crystallinity, mechanical strength, surface chemistry, degradation kinetics, porosity, drug release profiles, and cell–material interactions such as adhesion, infiltration, and immune modulation [14,15].

The impact of sterilization is especially pronounced in porous scaffolds, such as those fabricated through 3D printing or rapid prototyping, where finely tuned architectures are essential for function but inherently fragile. Distortions in scaffold geometry, pore collapse, or unintended acceleration of degradation can undermine surgical handling, compromise mechanical stability, and impair tissue integration. Achieving an optimal balance between scaffold porosity and degradation rate is critical for osteointegration, yet sterilization may disrupt this balance. Likewise, stimuli-responsive scaffolds—designed to undergo reversible changes in shape or volume—are particularly at risk, as terminal sterilization may induce permanent and undesirable modifications [16].

Traditional sterilization techniques remain widely used, but each presents significant limitations when applied to TERM-relevant materials. Steam sterilization, for instance, is effective and inexpensive but unsuitable for thermosensitive polymers, as exposure to heat and moisture can trigger hydrolysis and structural deformation. Ionizing radiation and EtO are common alternatives for heat-sensitive devices, yet both introduce critical drawbacks: radiation accelerates chain scission and polymer degradation, while EtO—though highly effective for complex or porous geometries—is toxic, flammable, and carcinogenic, with growing regulatory restrictions in both the EU and USA [8].

Together, these challenges underscore the urgent need to re-examine conventional sterilization strategies in the context of TERM, where maintaining the structural and functional integrity of advanced biomaterials is as critical as achieving sterility.

### 2.1. Steam and Dry Heat

Steam sterilization (moist heat) remains one of the most widely used methods in medical and laboratory practice due to its reliability, efficiency, and straightforward operation. Standard autoclave cycles expose devices to saturated steam at 121 °C for 15–20 min, although variations between 105 °C and 150 °C are possible depending on the material and bioburden level. Microbial inactivation occurs primarily through protein denaturation and membrane disruption, making this method highly effective against bacteria, spores, and viruses. The process typically involves a controlled sequence of pressurization, exposure, and depressurization, where careful regulation of heating and cooling rates minimizes mechanical stress from differential expansion [11].

Despite these advantages, moist heat is unsuitable for thermosensitive biomaterials. Degradable polymers such as PLA and PCL undergo hydrolysis and shrinkage, while protein-based hydrogels and collagen-rich scaffolds are prone to denaturation or swelling that disrupts pore architecture and compromises mechanical integrity [1]. In the context of TERM, such changes impair scaffold performance by reducing nutrient diffusion, altering degradation kinetics, and limiting cell infiltration.

Dry heat sterilization, by contrast, operates without moisture and relies on high temperatures (typically 160 °C for ≥2 h) delivered via conduction, convection, and thermal radiation. It is particularly suited for non-aqueous materials such as glassware, powders, oils, and certain polymers, and avoids risks of corrosion or swelling associated with steam. However, it requires significantly higher temperatures and longer exposure times than moist heat to achieve equivalent microbial inactivation, especially for resistant spores and prions [11]. Many commodity polymers—including LDPE, ABS, and polystyrene—undergo deformation or chemical degradation under these conditions, whereas silicone-based materials generally retain stability and are sometimes preferred when radiation or gas sterilization is impractical.

Overall, both moist and dry heat sterilization are largely incompatible with TERM applications. Their reliance on elevated temperatures and, in the case of steam, high moisture content, makes them unsuitable for most degradable polymers, proteinaceous scaffolds, and bio-responsive matrices that form the cornerstone of regenerative therapies.

### 2.2. Ionizing Radiation

Ionizing radiation, encompassing gamma rays, electron beams (E-beam), and X-rays, is a well-established terminal sterilization method widely applied to mass-produced medical devices due to its scalability and ability to sterilize prepackaged products. Microbial inactivation occurs through DNA damage induced by high-energy photons or electrons, which disrupts replication and metabolic function. Standard sterilization doses typically range between 15 and 35 kGy, with 25 kGy recommended by ISO guidelines for most medical devices [10]. Required doses, however, vary depending on the microbial burden, with viruses and spores generally requiring higher levels of exposure.

Gamma irradiation, commonly generated from cobalt-60, offers exceptional penetration and is therefore suited for sterilizing dense or bulky products. However, it also poses significant risks to material integrity, as high-energy photons can cleave polymer chains, induce crosslinking, and alter crystallinity. These changes manifest as reduced molecular weight, embrittlement, odor generation, discoloration, and loss of mechanical durability. Biodegradable polymers such as PLA, PCL, and PLGA are particularly vulnerable, exhibiting accelerated degradation and compromised mechanical strength. Biological matrices, including collagen or decellularized tissues, are equally susceptible, undergoing fragmentation or denaturation following exposure [17].

E-beam sterilization operates at energies between 5 and 10 MeV and doses of 10–60 kGy. While it delivers the same DNA-damaging effect as gamma irradiation, it has shorter exposure times and consequently induces less material degradation. However, its limited penetration depth reduces suitability for thicker or denser devices, unless higher doses or alternative methods are employed [8,16].

Radiation-induced damage is strongly dose-dependent: chain scission lowers molecular weight and accelerates biodegradation, whereas crosslinking can stiffen or embrittle scaffolds, both outcomes being detrimental for TERM applications [17,18]. Strategies such as irradiation in inert atmospheres (e.g., nitrogen) or modifications in polymer composition can partially mitigate these effects [11], but overall, ionizing radiation remains incompatible with many TERM-relevant biomaterials due to its profound impact on structural and biological performance.

### 2.3. Gas-Based Sterilization

EtO has been a cornerstone of medical device sterilization since the 1950s, accounting for nearly half of all sterilized products worldwide due to its versatility and effectiveness [8,10]. Operating at low temperatures (37–63 °C) and moderate humidity (40–80%), EtO penetrates deeply into porous structures, woven fabrics, and devices with complex geometries, making it especially valuable for sterilizing heat- and radiation-sensitive materials [11]. Standard sterilization cycles involve gas concentrations between 450 and 1200 mg/L, with exposure phases lasting 1–6 h depending on device design, though the full process—including preconditioning, aeration, and biological testing—may extend over several days [8].

EtO achieves sterilization by alkylating cellular macromolecules, including nucleic acids and proteins, thereby disrupting microbial replication [16]. A typical cycle comprises five stages: preconditioning and humidification, gas introduction, exposure, evacuation, and aeration. This controlled, multi-step process is generally considered gentle on polymers, as it avoids the chain scission and crosslinking associated with radiation or heat. Nonetheless, subtle structural changes have been reported in certain biomaterials, such as altered fiber morphology in electrospun PLA membranes [16]. The ISO 11135:2014 [19] standard provides detailed guidance on EtO sterilization, including requirements for process design, monitoring, and validation to ensure both sterility assurance and patient safety [10].

Despite its advantages, EtO use is increasingly restricted due to significant safety and environmental concerns. The gas is toxic, carcinogenic, and flammable when undiluted, with residual EtO posing risks of protein modification and DNA damage upon implantation [8,20]. Long processing times, diffusion limitations, and the need for extended aeration further reduce its economic efficiency [11]. These issues have prompted regulatory bodies such as the FDA to drive research into safer, more sustainable alternatives [5,6].

Among emerging options, hydrogen peroxide plasma and peracetic acid sterilization have gained attention. Both operate at lower temperatures and generate fewer material compatibility issues than steam or radiation. However, they present their own challenges, including hazardous waste generation, limited penetration into porous scaffolds, and material-specific interactions [2]. While promising, these alternatives remain constrained in their applicability to TERM materials, underscoring the need for continued innovation in gas-based sterilization strategies.

### 2.4. Low-Temperature Oxidative Sterilization: Hydrogen Peroxide Plasma and Ozone

To overcome the limitations of conventional sterilization methods, oxidative approaches such as hydrogen peroxide (H_2_O_2_) plasma, peracetic acid, and ozone have emerged as lower-temperature, residue-free alternatives suitable for heat- and moisture-sensitive materials. These agents are generally safe for human health but require careful handling and process design. For instance, concentrated hydrogen peroxide is highly reactive and hazardous, though it decomposes into water and oxygen post-sterilization. Ozone must be generated in situ and rapidly reverts to oxygen, providing an environmentally friendly sterilization route. Nevertheless, equipment and materials must be selected for oxidation resistance and compatibility with vacuum conditions, as these processes can otherwise damage sensitive biomaterials [10,16,18].

Plasma is a partially ionized gas containing charged and neutral particles, which makes it highly responsive to electromagnetic fields. Microbicidal plasmas are broadly classified into low-pressure and atmospheric-pressure types. Low-pressure plasmas exert their effects via ultraviolet photons, localized heat, and spore wall erosion, leading to DNA damage accumulation and impaired repair. Atmospheric-pressure plasmas primarily induce chemical and thermal disruption of microbial cell walls, proteins, and DNA, preventing replication [10]. While highly effective, plasma sterilization has practical limitations, including high cost, small chamber capacity, and extended treatment durations, restricting its scalability for large-volume sterilization.

Vaporized hydrogen peroxide (VHP), often combined with low-temperature plasma, has gained prominence as an alternative to ethylene oxide. H_2_O_2_ generates reactive oxygen species (ROS) that damage microbial proteins, lipids, and DNA, while the plasma phase decomposes residual peroxide into water and oxygen, minimizing toxic residues [11]. Typical operating temperatures range between 25 and 65 °C, making VHP compatible with many heat-sensitive polymers [16].

Despite its advantages, VHP faces important limitations. Penetration into dense scaffolds or long lumens is restricted, limiting sterilization efficacy. The requirement for vacuum conditions can deform packaging or delicate porous structures, and highly reactive ROS may oxidize sensitive biomolecules or surface chemistries. Additionally, plasma systems are costly and typically accommodate small batch sizes, limiting industrial-scale applications [10,16]. To address the penetration of VHP and the scalability limitations of plasma-based systems, gaseous ozone has emerged as a highly diffusive option for low-temperature oxidative sterilization. Although VHP struggles to permeate dense scaffolds or tortuous microchannels, ozone’s molecular weight and electronic structure enable deep penetration into intricate 3D biomaterial structures without the need for plasma-generating radiofrequency fields. Moreover, unlike costly plasma systems with limited chamber capacities, ozone can be generated in situ from air or pure oxygen to support large-volume sterilization workflows. Importantly for TERM applications, ozone spontaneously decomposes into molecular oxygen after treatment, minimizing the risk of cytotoxic chemical retention and supporting compatibility with subsequent cell-seeding processes. Nevertheless, VHP and plasma-based sterilization remain attractive for TERM applications because they avoid toxic residues, operate at low temperatures, and are generally less damaging to delicate biomaterials than irradiation or EtO.

The rationale for prioritizing ozone over other low-temperature oxidative sterilants is material- and geometry-dependent rather than absolute. Vaporized hydrogen peroxide (VHP) and H_2_O_2_ plasma are attractive because they operate at low temperatures and decompose to water and oxygen; however, they may show reduced penetration in long lumens, dense porous scaffolds, or highly tortuous architectures, and vacuum-assisted cycles can mechanically stress delicate hydrogel or fibrous constructs [4,10,16]. Peracetic acid is a strong broad-spectrum oxidant, but its use is more commonly associated with liquid or vapor chemical exposure and can require careful control of post-treatment residues or rinsing, particularly for absorbent or hydrated biomaterials [1,4,10,14]. Ozone occupies a distinct niche because it is generated in situ, decomposes rapidly to oxygen, can be delivered as a gas through open porous networks, and may simultaneously sterilize and mildly activate polymer surfaces by introducing oxygenated functional groups. For TERM, this combination is valuable when the target construct is acellular, thermosensitive, porous, and intended for subsequent cell seeding, provided that ozone dose, humidity, and exposure time are carefully optimized to avoid over-oxidation [2,6,21,22,23,24,25,26,27].

### 2.5. Emerging Technologies

Several emerging sterilization modalities aim to achieve effective microbial inactivation while minimizing damage to sensitive biomaterials. Supercritical carbon dioxide (scCO_2_) has attracted attention for its ability to sterilize under mild conditions, with tunable solvent properties that allow penetration into porous scaffolds. Despite these advantages, reliable microbial validation and standardization remain significant barriers to widespread implementation [1].

Cold atmospheric plasma is another promising approach, operating at near-ambient temperatures and producing reactive species and UV photons that inactivate microorganisms. While effective, its current applications are limited by small treatment volumes and challenges in achieving uniform sterilization across complex or dense structures [8].

Ozone sterilization is considered an environmentally friendly alternative. Because ozone is generated in situ and rapidly decomposes into molecular oxygen, it does not leave behind toxic gaseous residues. However, its strong oxidizing nature modifies material surfaces, leading to the formation of stable oxidation byproducts. The mechanistic pathway of this surface oxidation depends on the polymer’s chemical backbone. In unsaturated polymers containing carbon–carbon double bonds, ozone leads to hydrolytic chain scission and the generation of stable carbonyl and carboxyl groups. In contrast, it drives a free-radical autooxidation cascade in saturated polymers, via a hydrotrioxide intermediate, resulting in the formation of hydroperoxides, alcohols, carboxyls, and carbonyls [28].

In TERM, these chemical surface modifications prove highly beneficial. Through the introduction of polar, oxygenated functional groups (such as C–O, C=O, O–C=O, and –OH), ozone treatment markedly increases scaffold hydrophilicity. This enables the surface to better accommodate biomolecules (e.g., cell-adhesive proteins and growth factors), creating a thermodynamically inviting environment that helps cellular attachment and proliferation [29].

Ozone sterilization has emerged as an environmentally friendly alternative. Generated in situ, ozone rapidly decomposes to oxygen, leaving no toxic residues. It is highly effective against resistant microorganisms, including bacterial spores and biofilms. However, careful optimization of exposure parameters is critical, as oxidative stress can damage polymers and biomolecules. Additionally, ozone sterilization requires consideration of vacuum packaging and compatibility with oxidation-sensitive materials [10].

Other novel approaches, such as pulsed light and UV-C sterilization, are primarily suited for surface decontamination rather than volumetric sterilization. While promising for specific applications, their limited penetration restricts their utility for three-dimensional scaffolds or dense TERM constructs. Collectively, these emerging technologies illustrate a trend toward low-temperature, residue-free sterilization strategies that balance microbial inactivation with preservation of material integrity. However, each method currently faces practical, scalability, and validation challenges that must be addressed before routine clinical adoption.

### 2.6. Bridging Toward Ozone Sterilization

Despite decades of optimization, conventional sterilization methods remain fundamentally constrained for tissue engineering and regenerative medicine (TERM) applications. Heat-based approaches irreversibly denature proteins and distort polymer scaffolds, while ionizing radiation induces chain scission and oxidative fragmentation that compromise mechanical and biological properties. Ethylene oxide, although effective for complex geometries, carries significant toxicological risks and necessitates prolonged aeration periods. Hydrogen peroxide plasma, while cleaner and low-temperature, is limited by shallow penetration, small batch capacity, and high operational costs. Emerging technologies, including supercritical CO_2_ and cold plasma, show promise but face challenges in scalability, uniformity, and validated microbial inactivation.

These limitations underscore a persistent unmet need: sterilization strategies that reliably ensure microbial safety while preserving the structural, mechanical, and biological fidelity of advanced biomaterials. Ozone sterilization has recently emerged as a compelling alternative, offering broad-spectrum antimicrobial activity, rapid decomposition into oxygen, and environmentally sustainable operation under mild conditions. The following section critically examines ozone-based sterilization in the context of TERM, evaluating its advantages and challenges relative to conventional and emerging methods, and assessing its potential for clinical translation.

## 3. Ozone as a Sterilization Method

### 3.1. Ozone: Physical and Chemical Properties

Ozone (O_3_) is a triatomic allotrope of oxygen with a molecular weight of 48 g/mol, characterized by its high reactivity, which arises directly from its electronic and structural configuration. Its molecular structure, elucidated in 1952, is best described as a resonance hybrid consisting of four contributing resonance forms. This configuration results in equivalent O–O bond lengths that lie between the typical single and double oxygen–oxygen bonds. While the O–O single-bond length is 1.49 Å and the O=O double-bond length in molecular oxygen is 1.21 Å, the experimentally determined bond length in ozone is approximately 1.278 Å, consistent with the hybrid resonance model. The geometry of the molecule further contributes to its chemical behavior: although the expected O–O–O bond angle is 120°, experimental measurements indicate an angle of 116.78°, a deviation attributed to electron repulsion and charge distribution within the bent molecular configuration. These structural and electronic features explain ozone’s dual reactivity, enabling it to function as both an electrophilic and nucleophilic oxidant depending on the reaction environment and the nature of surrounding organic molecules [30].

Under standard conditions, ozone is a pale blue gas with a density of 2.14 g/L at 0 °C and 101.3 kPa—substantially higher than that of air (1.28 g/L). When cooled to its condensation point of −111.9 °C, ozone forms a dark blue liquid with a refractive index of 1.2226, and at its freezing point of −192.7 °C, it solidifies into a dark, almost black material. In atmospheric conditions, however, ozone appears colorless and exists as a mixture with O_2_ and N_2_ that behaves approximately as an ideal gas. Notably, ozone–air mixtures containing more than 10–11% ozone by volume become explosive due to the intense energy release associated with rapid ozone decomposition [31].

Ozone exhibits greater solubility in water than oxygen at temperatures between 0 and 30 °C, with solubility increasing at lower temperatures. It is even more soluble in non-polar organic solvents such as carbon tetrachloride. Ozone is inherently unstable, and its decomposition rate correlates strongly with temperature; at room temperature, it decomposes almost immediately, necessitating continuous generation to maintain stable concentrations [32]. The stability of ozone varies by phase: its half-life in the gaseous state is approximately 12 h, whereas in aqueous environments it is significantly shorter due to interactions with dissolved salts and impurities. In distilled water at 20 °C, the half-life ranges from 20 to 30 min and is reduced by several minutes in drinking water. Its decomposition is further influenced by pH, decreasing with increasing alkalinity; however, this trend is altered in the presence of certain additives, such as hydrochloric acid [32].

With an oxidation-reduction potential of 2.07 V, ozone is the fourth most powerful oxidizing agent known, enabling it to readily degrade organic compounds—including phenols and aromatic hydrocarbons—and to oxidize inorganic substances such as sulfurous and nitrous species. It also oxidizes metals like iron and manganese, even when they exist in complexed forms that would otherwise resist isolation [33]. While this strong oxidative capacity underlies ozone’s antimicrobial efficacy, it also accounts for its potential toxicity: exposure to concentrations above 100 ppb can damage mucosal surfaces and respiratory tissues in animals [31]. The balance between reactivity, instability, and environmental sensitivity defines ozone’s unique physicochemical profile and underscores the need for controlled conditions during its application in sterilization and biomedical contexts. Table 1 and Table 2 present the basic physicochemical properties of ozone and the oxidation–reduction potential of compounds and chemical elements, respectively.

### 3.2. Ozone Generation Methods and Their Effects on Biomaterials

**Figure 2:**
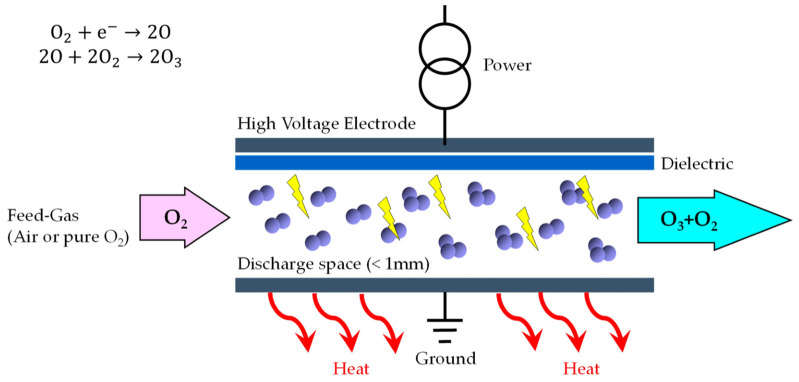
Schematic representation of ozone generation via the corona discharge method. Reprinted with permission from Ref. [34].

The choice of ozone generation method is an important parameter in the sterilization of tissue engineering scaffolds, as different systems produce distinct reactive species that interact uniquely with biomaterials. Corona discharge is the most widely adopted technique because it generates ozone by applying a high-voltage electrical field across an oxygen-containing gas stream, converting oxygen according to the reaction (Figure 2). While this method is cost-effective and capable of producing high ozone concentrations (3–6%), the use of atmospheric air can lead to the formation of nitrogen oxides (NOx) as undesirable byproducts. This can be minimized through air-drying systems or the use of pure oxygen concentrators.

**Figure 3:**
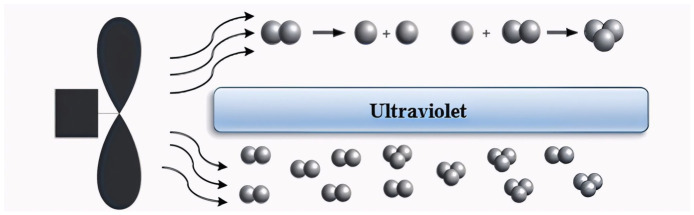
Schematic representation of ozone generation via ultraviolet (UV) light. Reprinted with permission from Ref. [35].

Alternatively, ultraviolet (UV) radiation, typically at wavelengths of 185 nm and 254 nm, generates ozone by photolyzing molecular oxygen (Figure 3). This method offers a cleaner oxidation environment since it does not produce harmful nitrogen byproducts. However, UV generation typically yields lower ozone concentrations (<0.5%) and requires longer exposure times to achieve effective sterilization.

Finally, Cold plasma systems generate ozone through dielectric discharge while simultaneously producing reactive oxygen species and highly reactive oxygen allotropes (e.g., O_4_ and O_5_). Although these species can lead to sterilization efficiency and enable rapid microbial inactivation, their oxidative nature may also intensify surface degradation of biomaterials. Therefore, their application in TERM necessitates optimization of exposure parameters to balance sterilization with the scaffold’s physicochemical properties.

### 3.3. Mechanism of Microbial Inactivation

The antimicrobial efficacy of ozone is primarily attributed to its strong oxidative potential, which enables it to react with a wide range of biological macromolecules. This pathway is typically surface-limited due to ozone’s high reactivity and limited penetration depth in dry or low-moisture conditions. Ozone readily oxidizes amino acids, leading to irreversible alterations in protein structure and function through denaturation. Amino acids particularly susceptible to oxidative attack include proline, histidine, sulfur-containing residues such as cysteine and methionine, and aromatic amino acids including phenylalanine, tyrosine, and tryptophan [36]. In addition to protein oxidation, ozone and the reactive oxygen species (ROS) generated during its decomposition can induce severe damage to nucleic acids. Unlike direct ozone attack, ROS-mediated oxidation can penetrate deeper into polymer matrices and contribute to bulk chain scission, crosslinking, or long-range structural changes. Free radical-mediated reactions result in DNA strand breaks, deformation of the double helix, and modifications of nitrogenous bases, ultimately impairing replication and transcription processes [37].

**Figure 4:**
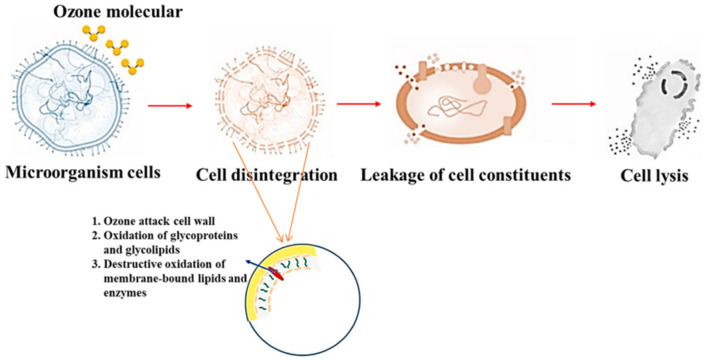
Schematic diagram of microbial inactivation by ozone. Reprinted with permission from Ref. [41].

Ozone generally acts initially on the outer components of microorganisms, oxidizing cell wall and membrane constituents before penetrating the cell and targeting intracellular components such as enzymes, proteins, DNA, and RNA. In bacteria, ozone initiates extensive lipid peroxidation via an electrophilic attack on carbon–carbon double bonds of polyunsaturated fatty acids [38]. This severe oxidation compromises membrane integrity, leading to increased permeability, leakage of cellular contents, and eventual cell lysis [39]. Compared to conventional disinfectants such as chlorine, ozone offers several advantages. Chlorine can form potentially harmful chlorinated organic by-products, whereas ozone rapidly decomposes into oxygen after application, minimizing environmental persistence. Moreover, ozone remains effective over a broad pH range, reducing the need for additional pH-adjusting chemicals in water treatment processes and thus representing an environmentally favorable alternative for microbial disinfection. Microorganisms exhibit varying levels of sensitivity to ozone exposure. In general, fungi demonstrate higher resistance than yeasts, while yeasts are more resistant than bacteria. Viruses exhibit sensitivity comparable to that of bacteria, although resistance increases in the presence of protective structures such as spores or complex cell envelopes [40]. Figure 4 illustrates the general schematic diagram of microbial inactivation by ozone treatment for the three categories of microorganisms analyzed: bacteria, viruses, and fungi.

Importantly, ozone should not be assumed to penetrate all microorganisms uniformly. Because gaseous and aqueous ozone are highly reactive, the initial lethal events are expected to occur predominantly at exposed cell-surface structures, including lipid membranes, envelope proteins, peptidoglycan, viral capsid or envelope proteins, and fungal cell-wall components [36,37,38]. For vegetative bacteria, secondary intracellular injury can occur after membrane disruption increases permeability, allowing ROS-mediated damage to enzymes and nucleic acids. For bacterial spores, however, direct penetration into the dehydrated spore core remains less certain. Electron-microscopy evidence indicates that ozone-treated Bacillus spores show marked disruption of the outer spore coat, suggesting that the coat and cortex are probable primary sites of ozone attack, while core inactivation likely depends on cumulative oxidative damage and hydration-assisted ROS formation rather than simple diffusion of intact ozone into the spore interior [21,38]. Therefore, this review interprets ozone sporicidal activity as a surface-initiated, humidity-enhanced oxidative process and not as unrestricted bulk penetration through microbial barriers.

#### 3.3.1. Deactivation of Bacteria After Ozonation

**Figure 5:**
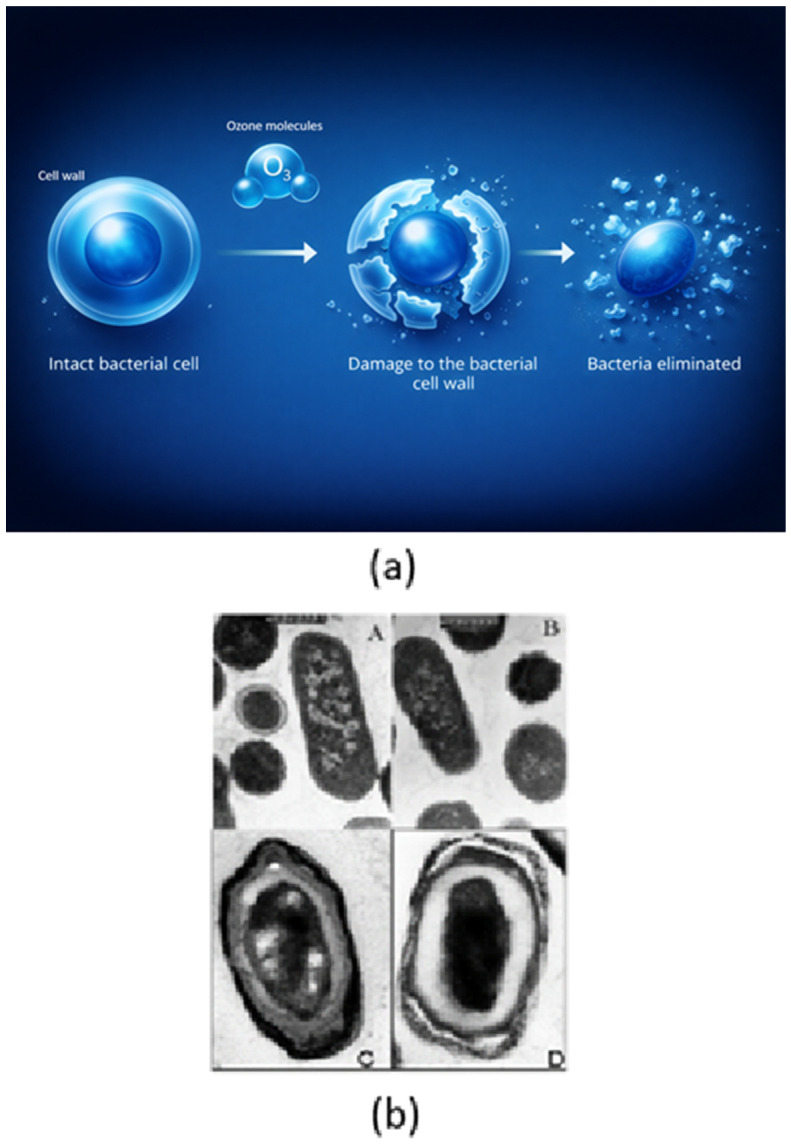
(**a**) Schematic representation of how ozone affects bacteria and eliminates them. (**b**) *Salmonella enteritidis* before (**A**) and after ozonation (**B**), and *Bacillus subtilis* before (**C**) and after ozonation (**D**). Reprinted with permission from Ref. [38].

Bacterial sensitivity to ozone varies according to structural and physiological characteristics. Gram-negative bacteria are generally more resistant to ozone than Gram-positive bacteria, while spore-forming bacteria exhibit significantly higher resistance compared to non-spore-forming species [32]. These differences are primarily attributed to variations in cell wall architecture and lipid composition. Ozone exhibits broad-spectrum antibacterial activity against both Gram-positive and Gram-negative bacteria through oxidative mechanisms. Its antimicrobial action is based on the oxidation of organic compounds, with bacterial susceptibility largely influenced by the composition and permeability of the cell envelope, as summarized in Figure 5.

The primary mechanisms of bacterial inactivation by ozone include [21,38,42]:

- **Cell wall and membrane disruption:** Ozone penetrates bacterial cell envelopes and oxidizes structural components. In Gram-positive bacteria, oxidation of the thick peptidoglycan layer compromises cell wall integrity, leading to cell lysis. In Gram-negative bacteria, ozone diffuses through the outer membrane and damages the thinner peptidoglycan layer, resulting in membrane destabilization;
- **Oxidative stress:** Ozone decomposition generates reactive oxygen species (ROS), including singlet oxygen, peroxide radicals, and hydroxyl radicals. These species induce oxidative stress by damaging lipids, proteins, and nucleic acids, ultimately leading to bacterial cell death;
- **Enzyme inactivation and metabolic disruption:** Ozone oxidizes key enzymes involved in cellular respiration and energy production, disrupting essential metabolic pathways and leading to irreversible loss of cellular function.
- **Spore coat disruption:** The absolute sterilization requires the inactivation of bacterial endospores, which is the definitive benchmark of microbial resistance. Ozone shows strong sporicidal activity by oxidizing the dense, proteinaceous outer spore coat. Once this structural barrier is dismantled, ozone penetrates the spore and irreversibly damages the inner membrane and core enzymes, inactivating the spore [43].

#### 3.3.2. Deactivation of Viruses After Ozonation

**Figure 6:**
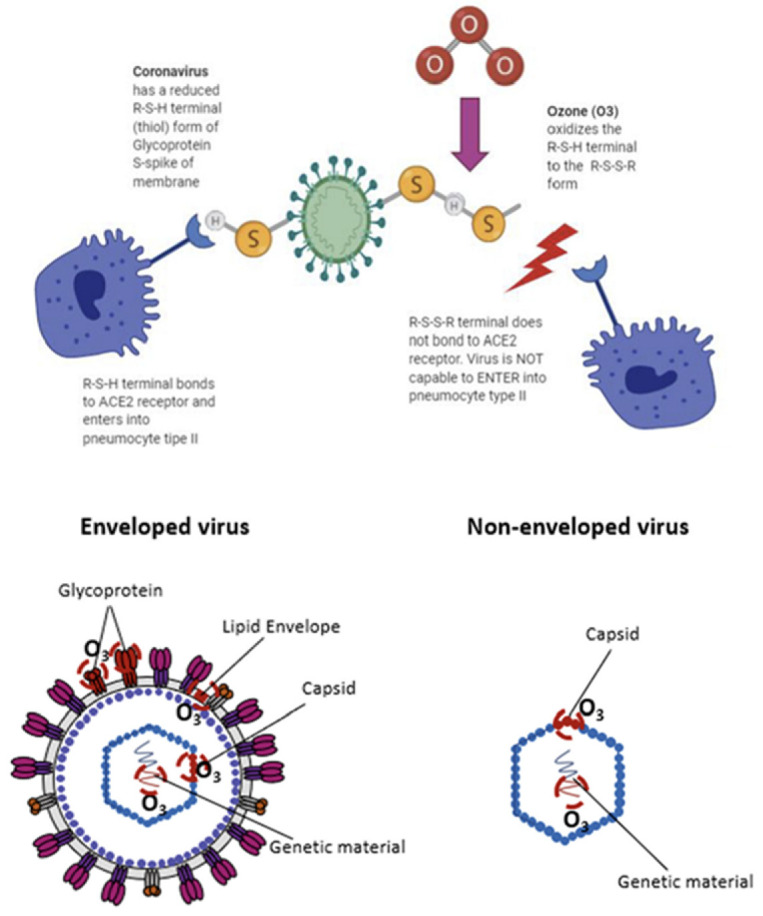
Mechanism of ozone in preventing the binding of S-spike protein residues to the ACE2 receptor and schematic representation of viruses with and without an envelope. Reprinted with permission from Ref. [52].

The inactivation of viruses by ozone has been less extensively studied compared to bacterial systems; however, existing research demonstrates that ozone is highly effective against a wide range of viruses. Viral inactivation typically occurs rapidly upon ozone exposure, although higher ozone concentrations are often required compared to those needed for bacterial inactivation [32]. Virus inactivation kinetics commonly exhibit a rapid initial decline in viral infectivity, often reaching reductions of up to 99%, followed by a slower phase requiring extended exposure times for complete inactivation. Viral sensitivity to ozone is strongly influenced by structural characteristics, particularly the presence or absence of a lipid envelope. Enveloped viruses are generally more susceptible to ozone than non-enveloped viruses due to the vulnerability of lipid membranes to oxidative damage. Unlike bacteria, viruses are not destroyed structurally but are rendered non-infectious. Ozone primarily inactivates viruses by oxidizing viral surface proteins and glycoproteins responsible for host cell attachment and entry. Oxidative modification of these receptors prevents viral binding to host cell membranes, thereby inhibiting infection and replication [44,45,46,47,48]. In addition to protein oxidation, ozone can damage viral lipids, lipoproteins, and nucleic acids, leading to further loss of viral infectivity. Enveloped viruses are particularly susceptible due to oxidation of envelope glycoproteins essential for receptor recognition and membrane fusion [49,50,51]. Figure 6 presents general schematic illustrations relevant to the antiviral action of ozone.

#### 3.3.3. Deactivation of Fungi After Ozonation

**Figure 7:**
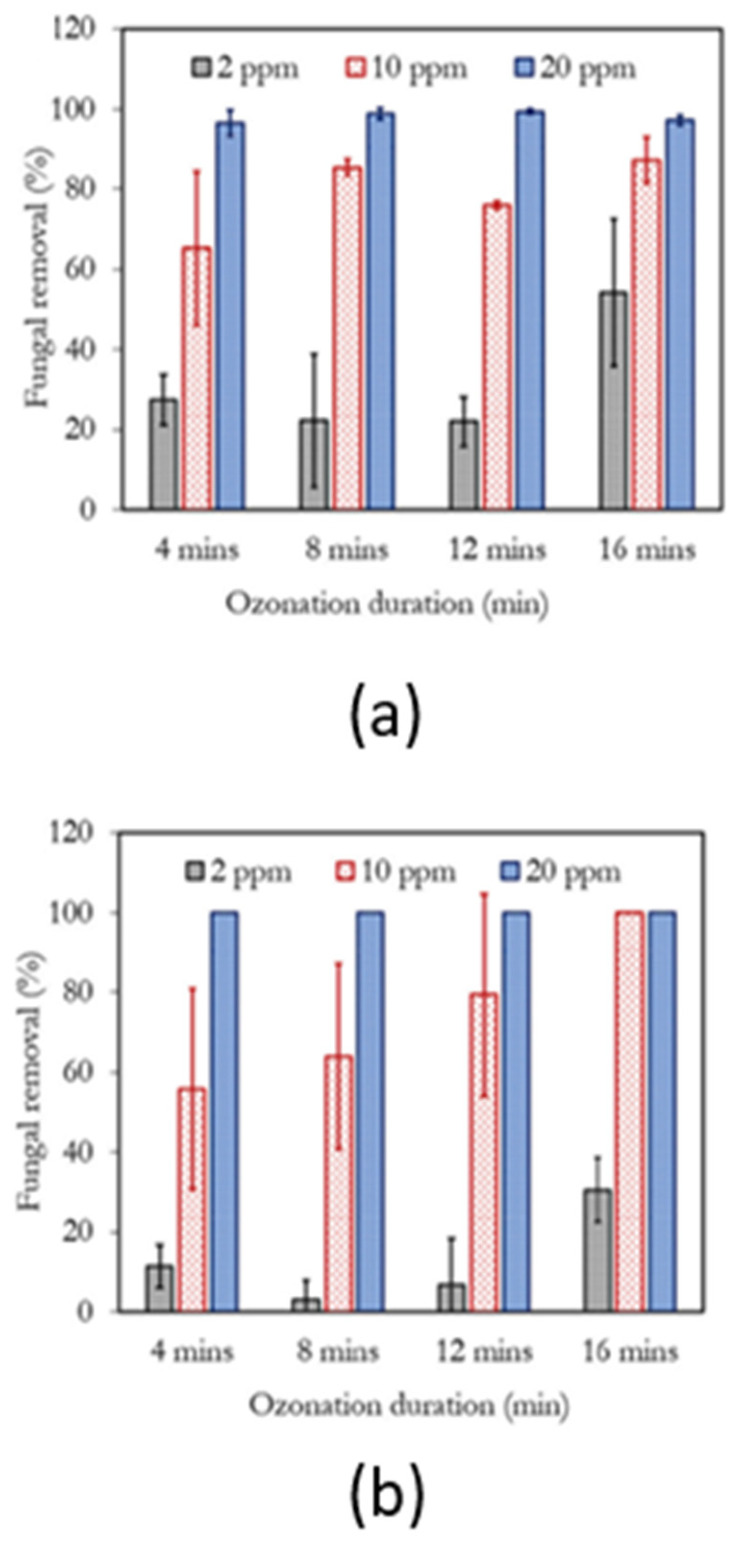
(**a**) Effect of ozone concentration in relation to exposure time on the elimination of *C. Albicans*; (**b**) Effect of ozone concentration in relation to exposure time on the elimination of *A. Fumigatus.* Reprinted with permission from Refs. [40,53].

Ozone is a strong oxidizing agent capable of inactivating fungi through multiple oxidative mechanisms, primarily targeting cell wall components, membrane lipids, proteins, and intracellular macromolecules. Fungal cells generally exhibit higher resistance to ozonation compared to bacteria, mainly due to their more complex and robust cell wall structure, which is rich in chitin, β-glucans, and mannoproteins, as well as the presence of spores in filamentous fungi. The effectiveness of ozone against fungal species, more specifically *Candida albicans* and *Aspergillus fumigatus*, is illustrated in Figure 7. As expected, increasing ozone concentration resulted in enhanced microbial reduction. However, both fungal species demonstrated significantly higher resistance to ozone exposure than the bacterial species tested under identical experimental conditions. At an ozone concentration of 2 ppm, *A. fumigatus* exhibited the highest resistance, showing only a 30% population reduction after 16 min of exposure, compared to a 54% reduction observed for *C. albicans*. This increased resistance of *A. fumigatus* has been previously reported by Hudson and Sharma (2009), who demonstrated that a single ozonation cycle was sufficient to inactivate *C. albicans*, whereas two cycles were required for effective decontamination of *A. fumigatus* [53]. The enhanced resistance of *A. fumigatus* is commonly attributed to its ability to form airborne conidia with thick protective layers, which limit ozone penetration and delay oxidative damage. At the maximum applied ozone concentration of 20 ppm, a short exposure time of 4 min was sufficient to achieve complete fungal inactivation for both species. To verify the completeness of *A. fumigatus* removal, treated samples were further incubated for an additional 24 h (72 h total incubation time). No fungal growth was observed in any of the dispersions following ozone exposure at 20 ppm, confirming the fungicidal efficacy of high-concentration ozonation under the studied conditions [40]. Overall, fungal deactivation by ozone is primarily associated with oxidative disruption of the cell envelope, increased membrane permeability, and subsequent damage to intracellular components, ultimately leading to loss of metabolic activity and cell death.

### 3.4. Kinetics of Disinfection

The kinetics of disinfection describe the rate of destruction of microorganisms by steam using a first-order chemical reaction rate model [54].

The kinetics of microbial inactivation by ozone are commonly described using first-order reaction models, in which the rate of microorganism destruction is proportional to the number of viable organisms remaining. This approach is widely applied to gaseous and aqueous ozone disinfection processes and forms the basis of the Chick–Watson kinetic model.

As the population of microorganisms (*N*) decreases over time, the inactivation rate can be expressed as:

(1) $$-\frac{dN}{dt}=\;k_{d}N,$$

where *N* is the number of viable microorganisms at time *t*, and *k_d_* is the ozone-dependent disinfection rate constant.

Integration of Equation (1) with the appropriate initial condition yields:

(2) $$N\left(t\right)=N_{0}e^{-k_{d}t},$$

where *N*_0_ represents the initial number of viable microorganisms prior to ozone exposure. Linearization of Equation (2) gives:

(3) $$ln\left(\frac{N\left(t\right)}{N_{0}}\right)=-k_{d}t$$

A plot of $\ln\left(\frac{N}{N_{0}}\right)$ versus time results in a straight line with a slope equal to −*k_d_*, allowing direct determination of the disinfection rate constant.

To bridge the gap between theoretical kinetics and practical sterilization parameters, microbial inactivation is typically expressed as the decimal reduction time (*D*-value). The *D*-value is the exposure time required to reduce the viable microbial population by 90%, which equates to a 1-log reduction, under defined conditions. Mathematically, it translates the natural logarithm disinfection rate constant ($k_{d}$) into a logarithm format [55]:

(4) $$D=\;\frac{2.303}{k_{d}}$$

By substituting this relationship, the classical microbial survival curve can be expressed in its standard base-10 logarithmic format [55]:

(5) $$log10\left(\frac{N}{N_{0}}\right)=-\frac{t}{D}$$

From this relationship, the total exposure time required to achieve a 6-log reduction (99.9999% microbial inactivation) can be calculated as:

(6) $$t_{6-log}=6D$$

Furthermore, while traditional Z-values are used in thermal sterilization to describe the temperature change needed to reduce the *D*-value by a factor of 10, ozone kinetics rely on concentration dependence rather than temperature. Consequently, in ozone-based disinfection, the rate constant $k_{d}$ is not governed primarily by temperature, as in thermal sterilization, but depends on several operational parameters, including ozone concentration, exposure time, relative humidity, and the physicochemical properties of the treated medium or surface. The effect of ozone concentration is often incorporated using the concentration–time (CT) concept, expressed as:

(7) $$ln\left(\frac{N}{N_{0}}\right)=-kC^{n}t,$$

where *C* is the ozone concentration, *t* is the exposure time, *k* is an empirical kinetic constant, and *n* is an experimentally determined coefficient reflecting microorganism sensitivity to ozone.

This kinetic framework highlights the importance of optimizing ozone dose and exposure conditions to achieve effective microbial inactivation while minimizing potential oxidative damage to biomaterials, a critical consideration for sterilization strategies in tissue engineering and regenerative medicine applications.

Although ozone sterilization is frequently described as selective toward microbial inactivation over bulk material degradation, direct comparative activation-energy datasets for TERM-relevant biomaterials remain largely unavailable. As discussed previously, ozone rapidly inactivates microorganisms through oxidative disruption of membrane lipids, proteins, and nucleic acids [21,36,37,38,39,40,41,42], whereas polymer degradation mechanisms, including chain scission, oxidation, depolymerization, and alterations in crosslinking density, depend strongly on polymer chemistry, exposure duration, humidity, and ozone concentration [1,5,6,11,56]. Current evidence suggests that microbial inactivation generally occurs under milder exposure conditions than those required to induce substantial structural degradation in several ozone-compatible polymers, including PCL, PMMA, PE, and PEEK [5,6,11]. Nevertheless, systematic quantitative comparisons between microbial death kinetics and biomaterial degradation kinetics remain insufficiently explored. Consequently, further mechanistic studies are needed to establish whether ozone can consistently achieve sterility assurance while preserving the physicochemical and mechanical integrity of sensitive TERM scaffolds.

For reproducible TERM sterilization, ozone protocols must report at minimum ozone concentration, exposure time, relative humidity, temperature, pressure or vacuum profile, chamber volume, load mass, scaffold geometry, packaging, and the position at which ozone concentration is measured. Reported low-concentration laboratory decontamination studies commonly use ozone levels in the 2–20 ppm range for minutes-scale exposure; for example, fungal inactivation studies demonstrated concentration-dependent killing at 2, 10, and 20 ppm, with complete inactivation of C. albicans and A. fumigatus after short exposure at 20 ppm under the tested conditions [40,53]. By contrast, ozone gas sterilization validation studies using *Geobacillus stearothermophilus* biological indicators have examined much higher ozone concentrations, commonly in the 3000–15,000 ppm range, at 80–90% relative humidity and 25–35 °C, reflecting the substantially greater resistance of bacterial spores and the need for validated SAL-oriented cycles [57]. These values should be viewed as representative process windows rather than universal TERM recommendations, because the required dose depends strongly on the microbial challenge, scaffold porosity, material ozone demand, packaging, and target sterility assurance level.

Relative humidity is a critical process variable. Dry ozone is generally less effective against spores because limited water availability reduces microbial hydration, restricts ROS formation, and may prevent ozone-derived radicals from reaching sensitive cellular targets. Higher humidity promotes swelling/hydration of microbial envelopes and enhances formation of secondary oxidants such as hydroxyl radicals, thereby lowering D-values in ozone gas sterilization studies [21,57]. For TERM applications, an RH range of approximately 50–90% should be investigated during cycle development, with 80–90% RH representing a common high-humidity validation window for resistant spore indicators; however, hydrated scaffolds and hydrogels may require lower humidity or shorter exposure to prevent polymer swelling, hydrolysis, or loss of mechanical integrity.

Ozone instability must also be treated quantitatively. In a closed chamber without continuous ozone generation, ozone concentration decays with time according to chamber temperature, humidity, gas-phase decomposition, surface reactions, and ozone demand imposed by the load. Therefore, the delivered dose is better represented by the time-integrated ozone exposure, i.e., the area under the concentration–time curve, rather than by the nominal initial concentration alone. When ozone is continuously generated or pulsed, the process should be described by a mass-balance profile that includes generation rate, leakage or venting, decomposition, catalytic destruction, and consumption by the biomaterial surface. For porous TERM constructs, ozone concentration should ideally be monitored or validated at the most difficult-to-sterilize location, because chamber setpoint concentration may overestimate the dose delivered inside dense scaffolds, lumens, or packaged loads [6,31,32,54,57].

#### Comparative Inactivation Kinetics of TERM-Relevant Microorganisms

Establishing quantitative kinetic parameters for common biomaterial-associated pathogens is essential for standardizing ozone sterilization in TERM applications. Vegetative bacteria commonly implicated in these infections (e.g., Staphylococcus aureus and Pseudomonas aeruginosa) have high susceptibility to oxidative degradation and possess low kinetic resistance compared to endospores. For instance, the literature shows that a 6-log reduction in these microorganisms can be achieved within 20 min of exposure to gaseous ozone at a peak concentration of 25 ppm [58].

In contrast, bacterial endospores like *Bacillus subtilis* display profound kinetic resistance due to their protective proteinaceous outer coat, which acts as a reactive barrier against ozone. Once this outer barrier is structurally compromised, ozone irreversibly damages the inner membrane, oxidizing membrane proteins and unsaturated fatty acids to induce cell lysis [43].

### 3.5. Validation, Regulatory, and Safety Considerations for Ozone Sterilization in Tissue Engineering and Medical Devices

Ensuring sterility is fundamental to the safe and effective use of medical devices, particularly in surgical and other high-risk clinical procedures, where failures in cleaning, disinfection, or sterilization can lead to severe patient harm, elevated healthcare costs, and occupational hazards for healthcare personnel [59]. Consequently, regulatory authorities impose stringent frameworks to minimize contamination risks. In the European Union, the Medical Device Regulation (MDR 2017/745) and the In Vitro Diagnostic Regulation (IVDR 2017/746) mandate comprehensive sterility controls, while standards such as EN 556-1:2024 [60] specify the criteria under which devices may legitimately be labeled “sterile” [61]. Compliance with these requirements depends on robust quality assurance and quality management systems that document and control each phase of sterile processing. Central to this compliance is the sterilization process validation—including installation qualification (IQ), operational qualification (OQ), performance qualification (PQ), and microbiological performance qualification (MPQ)—all of which must demonstrate the reproducible achievement of critical process parameters across multiple successful cycles [59].

Microbiological validation further requires biological indicators containing highly resistant microorganisms to ensure a stringent challenge. For high-risk products such as implantable devices, post-sterilization culturing must confirm the complete absence of viable organisms [10]. Regulatory authorities also distinguish between sterilization technologies with established consensus standards (Class A) and those lacking such standards (Class B). Class A methods, which are gamma irradiation, electron beam irradiation, steam, dry heat, and ethylene oxide (EtO), are supported by ISO or equivalent third-party standards and are generally considered compliant when these standards are followed. In contrast, Class B methods, such as hydrogen peroxide gas plasma and ozone, do not have recognized consensus standards and therefore require expanded FDA review, more extensive documentation, and detailed validation protocols during premarket submissions [16]. These differences are summarized in Table 3.

Medical devices themselves are stratified by infection-related risk [62]—from Class I (low risk) to Class III (high risk)—with stringent sterility demands for higher-risk categories. Sterility Assurance Level (SAL) defines the probability that a single viable microorganism survives a sterilization process, expressed as 10^−n^ [11]. The universally adopted SAL of 10^−6^, required for Class III implantable devices under EN 46001 [63], reflects the expectation that no more than one non-sterile item may exist per million sterilized units [16]. Lower-risk devices may require an SAL of 10^−3^ [64]. Importantly, recent studies demonstrate that ozone sterilization can achieve an SAL of 10^−6^ under appropriate conditions, meeting the threshold for high-risk medical devices [40].

Applying these regulatory principles to tissue-engineered constructs introduces additional complexity. Scaffolds, hydrogels, nanofibrous matrices, and 3D-printed biomaterials are highly sensitive to heat, ionizing radiation, and other conventional sterilization processes, which may alter chemical structure, mechanical integrity, or pore microarchitecture. Although EtO offers compatibility with thermosensitive materials, its limitations include toxic residuals, long aeration times, and growing regulatory scrutiny. As a result, no universal sterilization approach exists for tissue engineering, and tailored validation studies are required to meet SAL thresholds while preserving material functionality [1]. Ozone presents several advantages—low-temperature operation, potent antimicrobial activity, and rapid decomposition to oxygen without leaving toxic residues—yet its regulatory acceptance remains limited. Classified as a non-traditional sterilization technology, ozone lacks FDA-recognized standards and long-term clinical validation, creating significant barriers to widespread clinical translation [25]. Current validation efforts therefore rely on ANSI/AAMI/ISO 14937:2009 [65], which governs novel sterilization modalities and requires rigorous characterization and control of parameters such as ozone concentration, humidity, exposure duration, and biological indicator resistance.

To validate post-treatment biocompatibility under these regulatory frameworks, evaluation protocols must align with the ISO 10993 [66] series. In particular, ISO 10993-5 [67] (in vitro cytotoxicity assays) is necessary to confirm that the ozone process does not produce cytotoxic polymer residues or toxic chemical residuals. Parallel evaluations according to ISO 10993-18 [68] (chemical characterization of materials) and ISO 10993-11 [69] (systemic toxicity) are equally important to screen for harmful degradation extractables. The combination of ISO 14937 [65] microbial inactivation parameters to achieve a verified SAL of 10^−6^ with ISO 10993 [66] biological screening, the researchers can establish a standardized testing pipeline that guarantees both sterility and patient safety for ozone tissue scaffolds.

To support researchers and manufacturers, the most relevant EN, ISO, and regulatory standards that apply to sterilization, biocompatibility assessment, quality management, and biological evaluation of materials—including those used in tissue engineering—are summarized in Table 4. Collectively, these standards highlight both the structured regulatory environment governing sterilization and the current gaps that hinder the broader clinical integration of ozone-based technologies.

Biological-indicator validation requires particular caution for ozone. Transferability from steam, ethylene oxide, or hydrogen peroxide cycles should not be assumed because BI resistance depends on the sterilant, carrier material, packaging, humidity, temperature, and local ozone accessibility. *Geobacillus stearothermophilus* spores, including ATCC 12980 and ATCC 7953, have been used as ozone biological indicators, but their D-values differ substantially depending on strain and carrier chemistry; in one ozone gas study, the CT resistance of G. stearothermophilus ATCC 12980 was approximately twice that of ATCC 7953, and higher humidity, ozone concentration, and temperature reduced D-values [57]. Accordingly, TERM validation should use ozone-calibrated biological indicators or product-specific inoculated carriers placed in worst-case scaffold locations, including internal pores, lumens, hydrated regions, and packaged areas with limited gas exchange. For implantable TERM constructs, BI testing should be combined with bioburden assessment, sterility testing, extractables/leachables analysis, cytotoxicity testing, and post-sterilization functional assays.

Oxidative sterilants may also generate sublethally injured microorganisms that are initially non-detectable under selective or short incubation conditions but recover during storage or after transfer to favorable media. This issue is particularly relevant for ozone because membrane oxidation, protein damage, and DNA injury may be incomplete at insufficient CT values. To reduce the risk of false-negative sterility conclusions, validation should include recovery on non-selective media, comparison with selective media when appropriate, extended incubation or delayed enumeration, and post-treatment hold-time studies under storage conditions relevant to the sterilized scaffold or device [70]. These controls are especially important for TERM materials containing proteins, polysaccharides, or hydrated polymer networks that may partially protect microorganisms or consume ozone before complete inactivation is achieved.

**Table 4:** Standards and Regulations Relevant to Sterilization in Tissue Engineering.

Standard/Regulation	Organization	Application	Notes
MDR (EU) 2017/745 [71]	European Union (EU)	Medical devices regulation	Control of contamination, infection prevention, and sterility for medical devices.
IVDR (EU) 2017/746 [72]	European Union (EU)	In vitro diagnostic medical devices	Similar to MDR, it is applied specifically to diagnostic devices.
EN 556-1 [60]	European Committee for Standardization (CEN)	Sterile labeling	Requirements for a device to be legitimately labelled as “sterile”.
EN 46001 [63]	European Committee for Standardization (CEN)	Quality system for implantable devices	Requires a SAL of 10-6 for high-risk Class III implantable devices.
ISO 10993 Series [66]	ISO	Biocompatibility assessment	Biological evaluation. Recognized by FDA and EU regulators.
ISO 14937 [65]	ISO	Novel sterilization methods	General framework or validation of non-traditional methods (including ozone).
Regulatory Gap for Ozone Sterilization	Pending	Ozone Sterilization in Tissue Engineering	Absence of ozone standards or FDA guidance.
ASTM D1149 [73]	ASTM International	Ozone resistance testing of polymers and elastomers.	Standard methods for polymer resistance and surface cracking under ozone environments.

#### 3.5.1. Risk Categorization and Laboratory/Clinical Safety Considerations

Risk categorization extends to laboratory and clinical settings, where the level of device contact with the human body dictates required decontamination. Critical devices—such as implants, surgical instruments, and endoscopes—must undergo thorough cleaning followed by terminal sterilization. Semi-critical devices contacting mucous membranes or non-intact skin should be sterilized whenever possible, while non-critical devices require cleaning and low- to intermediate-level disinfection [61]. Ozone sterilization may be used for critical devices only when manufacturers confirm material compatibility and the ozone sterilizer carries appropriate regulatory approvals (FDA or CE), as summarized in Table 5.

Despite its advantages, ozone carries inherent safety considerations. As a strong oxidant, it is an air pollutant and respiratory irritant associated with reduced lung function, airway inflammation, and increased susceptibility to respiratory complications even at low exposure levels. In vitro studies demonstrate that ozone concentrations around 1.5 ppm can impair epithelial cell function—for example, reducing CFTR expression—and interact with skin lipids to generate secondary irritants [74]. Accordingly, ozone sterilization rooms must remain unoccupied, sealed, and free of flammable materials during operation. Personnel must verify safe equipment setup, ensure grounded power systems, and use appropriate personal protective equipment (PPE), including masks, gloves, and goggles. Training in emergency response—especially for potential ozone leaks—is essential, as is proper disposal of ozone-exposed materials. Ultimately, successful integration of ozone sterilization in tissue-engineering workflows requires carefully balancing sterilization efficacy, material compatibility, operator protection, and regulatory compliance.

#### 3.5.2. Health and Safety Considerations

Ozone’s powerful antimicrobial nature necessitates careful containment to protect operators, although it decomposes rapidly to oxygen [5,6]. Workplace exposure must be controlled below 0.1 ppm (8 h TWA) using ozone destruction units and adequate ventilation. Unlike EtO, ozone leaves no residual chemicals on sterilized products [2]. OSHA sets a short-term exposure limit (STEL) of 0.3 ppm over 15 min and a permissible exposure limit (PEL) of 0.1 ppm (8 h TWA), while NIOSH recommends a maximum of 0.1 ppm and classifies concentrations ≥ 5 ppm as Immediately Dangerous to Life or Health (IDLH). ACGIH similarly recommends ceilings of 0.1–0.3 ppm for short-term exposures [4]. Notably, humans can detect ozone at 0.003 ppm, providing early warning.

Ozone exposure can also originate outside sterilization chambers, such as from air purifiers, photocopiers, or laser printers. Poorly ventilated spaces may see concentrations rise to 0.5 ppm if filtration and monitoring are inadequate (WorkSafeBC). Modern ozone sterilizers often operate in negative-pressure chambers to minimize leakage and facilitate rapid decomposition to oxygen. Manufacturers recommend at least ten air exchanges per hour to ensure residual ozone clearance. Effective sterilization requires optimizing operational parameters such as exposure dose (concentration × time), temperature, and relative humidity to balance microbial inactivation and occupational safety [75].

#### 3.5.3. Environmental Considerations

Ozone is a potent oxidant capable of inactivating pathogens in water, pharmaceuticals, food, and medical device applications. Its rapid decomposition into oxygen and absence of persistent residues make it an environmentally sustainable alternative to traditional disinfectants, which can generate toxic by-products or require high-energy processes [36,40]. Nevertheless, ozone at ground level is a harmful atmospheric pollutant formed via photochemical reactions between NO_x_ and VOCs, contributing to smog and respiratory health risks [59]. Regulatory frameworks limit ambient exposure: the EU Ambient Air Quality Directive (2008/50/EC) caps the maximum daily 8 h mean at 120 µg/m^3^, and the WHO recommends 100 µg/m^3^ [59]. In controlled sterilization, however, on-site ozone generation and its short half-life mitigate these environmental and safety concerns [4].

In summary, ozone sterilization offers a promising solution for sterilizing both conventional and highly sensitive tissue-engineered medical devices, combining potent antimicrobial activity with low-temperature operation and rapid decomposition to oxygen. However, its broader clinical implementation depends on navigating a complex landscape of regulatory requirements, rigorous validation protocols, and occupational safety standards. Achieving the required Sterility Assurance Levels while preserving material functionality necessitates careful optimization of process parameters, comprehensive microbiological validation, and strict adherence to quality management systems. Concurrently, operator and environmental safety must be ensured through engineering controls, exposure monitoring, and adherence to established occupational and ambient ozone limits. While current standards and regulatory guidance provide a framework, continued research, standardization, and harmonization are essential to fully integrate ozone sterilization into routine medical device and tissue engineering workflows, maximizing both patient safety and material efficacy.

## 4. Compatibility of Ozone with Biomaterials

Maintaining sterility is essential for biomaterials intended for implantation or close contact with living tissues, including organs, skin, and bone. However, conventional sterilization methods can subject materials to physical and chemical stresses, potentially altering their composition, microarchitecture, mechanical properties, or even generating toxic by-products [1]. Therefore, selecting an appropriate sterilization method requires careful consideration of the material’s chemical composition, structural features, and intended biomedical application to ensure both safety and functionality.

Ozone has emerged as a promising sterilization modality in tissue engineering due to its strong oxidative potential, which enables rapid microbial inactivation. Additionally, ozone decomposes naturally into oxygen and leaves minimal toxic residues, offering an environmentally friendly alternative to traditional sterilants. Nonetheless, ozone’s reactivity can induce undesired effects, including depolymerization, dereticulation, or oxidative degradation via free radical generation [56]. Understanding ozone–material interactions is therefore critical for evaluating their impact on mechanical integrity, surface chemistry, and biocompatibility across a range of biomaterials, including polymers, metals, ceramics, and composites.

### 4.1. Interactions with Polymers, Metals, Ceramics, and Composite Scaffolds

Effective ozone sterilization requires high humidity (>80%) to facilitate microbial inactivation. However, this combination of humidity and oxidative reactivity can create compatibility challenges, particularly for polymeric and composite scaffolds. Polymers exhibit the broadest range of responses: their oxidative resistance and structural stability under humid ozone conditions depend on chemical composition and surface hydrophilicity. Hydrophilic coatings may swell or lose functionality, whereas non-hydrophilic coatings better retain structural and functional integrity [11].

High-resistance polymers such as polyethylene (PE), polypropylene (PP), polyallomer (PP/PE blend), polycarbonate (PC), polyether ether ketone (PEEK), and fluoropolymers (PTFE, PVDF, ETFE, FEP) demonstrate minimal degradation under ozone exposure. Fluoropolymers, in particular, resist oxidative damage due to their chemical inertness and strong carbon–fluorine bonds, making them suitable for long-term biomedical applications, including vascular grafts and joint prostheses. Polyethylene and polypropylene maintain mechanical and morphological stability after repeated ozone sterilizations, with low- and medium-density PE showing slightly higher oxidation resistance than high-density forms. Polycarbonate retains transparency and mechanical strength, and mild oxidation may even enhance surface energy. PEEK exhibits robust structural integrity after multiple ozone cycles, rendering it ideal for reusable instruments and tissue-engineered scaffolds [11].

Polymers with moderate resistance, like polysulfone (PSF), polyphenylsulfone (PPSU), polyhydroxybutyrate (PHB), polyamide (nylon), poly(methyl methacrylate) (PMMA), and polyvinyl chloride (PVC), generally tolerate controlled ozone exposure. PSF may undergo mild surface embrittlement over prolonged treatment, and polyamides are more sensitive to chain cleavage. PVC is stable under moderate ozone exposure, though minor discoloration and surface gloss loss may occur [11].

Hydrogels present unique challenges due to high water content and soft mechanical properties. Effective sterilization must preserve the hydrogel structure and function while eliminating microbial contamination. Drying before sterilization is sometimes employed to reduce degradation, but this is not feasible for applications such as contact lenses, drug delivery systems, or bioinks. Silicone hydrogels maintain functionality at low ozone concentrations, though higher doses increase ionic permeability and reduce mechanical stability. Chitosan hydrogel nanoparticles largely retain structural properties, although ozone is less effective than gamma irradiation and may induce mild toxicity. Protective sugars in hydrogels can undergo chemical modifications during ozonation [1].

Polyesters maintain their macrostructural stability during ozone exposure because their saturated carbon backbones and stable ester bonds lack electrophilic targets for ozonolysis, making degradation kinetics insignificant. Consequently, controlled ozone treatment of Polylactic acid (PLA) and Polycaprolactone (PCL) frameworks has been shown to produce no meaningful loss of tensile strength [76], while spectroscopic analyses (FTIR and Raman) verify that the chemical integrity and physical microstructure remain unaltered [77].

In addition, polyethers are moderately susceptible to ozone degradation through hydrogen abstraction at methylene groups, displaying first-order kinetic dependence on ozone concentration [78]. Although ozone attack is kinetically restricted under dry or low-pH conditions, the degradation process accelerates dramatically in high humidity or high pH. In these humid sterilization environments, ozone decomposes into highly reactive hydroxyl radicals that induce rapid chain scission and molecular weight decay.

Polysaccharides follow a different degradation path that is governed by an indirect radical oxidation, where reactive hydroxyl radicals are released during ozone decomposition in water. This radical pathway targets the glycosidic linkages of large polymer chains, causing a fast decrease in both molecular weight and viscosity over time. Notably, because ozone targets larger macromolecules at a higher rate, it yields a highly desirable, narrower, and more uniform molecular weight distribution across the material [79].

Low-resistance polymers—including polystyrene (PS), polyurethane (PU), butyl and natural rubber, and polychloroprene—are generally unsuitable for ozone sterilization due to rapid oxidative degradation, with unsaturated polymers being particularly vulnerable [5,6]. Cellulosic materials experience partial oxidation and decreased mechanical strength, though derivatives such as cellulose acetate show improved resistance.

Metals and ceramics typically exhibit higher ozone stability due to dense atomic structures. Stainless steel (grades 304 and 316) and titanium remain structurally and morphologically intact after repeated exposure, with surface oxidation potentially enhancing chemical reactivity and osteointegration [5,6,17]. However, catastrophic phenomena related to environmentally affected structural flaws under fatigue [80] or to “hydrogen-like” absorption [81], should not be excluded and need further investigation. Conversely, reactive metals such as zinc and cast iron undergo corrosion under ozone exposure, while the stability of aluminum depends on dry versus wet conditions [5,6]. Composite scaffolds exhibit variable ozone tolerance, influenced by polymer matrix, filler composition, and interfacial stability.

Table 6 summarizes the compatibility of polymers, metals, and composites with ozone sterilization, providing a useful reference for choosing materials in tissue engineering and regenerative medicine applications.

### 4.2. Impact on Mechanical Integrity, Surface Chemistry, and Bioactivity

For biodegradable scaffolds, the sterilization method must maintain the structural and biochemical properties to ensure consistent post-sterilization; however, it is often challenging to achieve this [14,82]. Ozone is generally considered a compatible sterilizer for many polymers, but its effectiveness depends on many parameters, like temperature, concentration, and humidity. Changes in these parameters can affect oxidation rates, leading to alterations in the surface chemistry and mechanical properties of the material [11].

The mechanical behaviour of polymers to ozone also depends on chemistry and the duration of exposure. For example, a study on a long-term ozone exposure of a high-temperature epoxy showed that ozone penetrated up to 120 μm below the surface, demonstrating that ozone can affect not only surface layers but also deeper regions of a polymer. More specifically, initial crosslinking reactions increased stiffness, but long ozone exposure led to chain scission and material weakening. This behavior shows that ozone degradation is complex and not always progressive [5,6].

The characterization of ozone sterilization as a “gentle” approach depends strongly on the biomaterial type and exposure conditions. While synthetic polymers such as PCL and PMMA may retain morphology and mechanical stability under controlled ozone exposure [5,6,11], hydrogel-based systems including alginate- and gelatin-derived materials are generally more sensitive to oxidative modifications that may alter swelling behavior, crosslinking, and bioactivity [1].

The adjustment of environmental conditions can limit some of these effects. For example, cooling the environment to below ambient (but above freezing) can improve ozone stability and decrease the required dosage for sterilization, but it may also require longer exposure times. Conversely, lowering ozone concentration and temperature can mitigate its effect on polymers. Thus, it is important to optimize operational parameters to achieve a balance between sterilization efficiency and material preservation [11].

Ozone modifies scaffold morphology, microstructure, and mechanical behavior. For instance, ozone sterilization of polyurethane (PU) foams has been shown to create a new population of smaller pores and increase pore interconnectivity. These alterations modify the scaffold’s roughness and wettability, which in turn can impact cell adhesion and overall cell-material interactions [56]. The mechanical response to ozone exposure, however, is different for every polymer type. For example, polyhydroxybutyrate (PHB) shows increased elasticity and modulus after ozonation, whereas silicone hydrogels experience losses in strength and elongation at higher ozone doses [1,56].

Although material properties naturally react differently to ozone, many contrasting results in the literature are often caused by unstandardized experimental protocols rather than material differences alone. An important limitation in many studies is the failure to characterize operational parameters, like ozone concentration (ppm), gas flow rate, and environmental humidity. Additionally, discrepancies in mechanical stability are often affected by the scaffold’s architecture. For example, highly porous structures possess a much larger surface area-to-volume ratio than dense bulk films, making them far more vulnerable to oxidative degradation despite being fabricated from the same polymeric material. Furthermore, the timeline of evaluation must be considered. The assessment of mechanical properties immediately after sterilization overlooks how hydrophilic oxidation byproducts can alter water absorption and accelerate long-term hydrolytic degradation in vivo. These methodological gaps show the need to control ozone concentration, exposure time, humidity, and pH to avoid unwanted material degradation.

Moreover, even when experimental conditions are perfectly standardized, fundamental mechanical variations persist among different biomaterials. This is explained by the chemical selectivity of the oxidant and mass transport limitations. Whereas ozone specifically targets the unsaturated carbon–carbon double bonds in microbial membranes to induce lipid peroxidation and cell lysis (as discussed in Section 3.2), many hydrogel matrices are composed of saturated polymer backbones and stable crosslinking junction zones. Since these hydrogel networks lack reactive aliphatic double bonds, they remain chemically inactive during exposure. Furthermore, the high macromolecular density of a macromolecular scaffold creates mass transport resistance. As a result, it causes the outer boundary layer to act as a sacrificial oxidative shield that limits deep gas diffusion and safeguards the internal bulk crosslinking network.

The maintenance of sterility without compromising the microstructural and physicochemical properties of scaffolds remains an important challenge [56]. The oxidative modifications by ozone can change surface charge and hydrophilicity, affecting bioactivity, protein adsorption, and cell affinity. This occurs because ozone-induced surface oxidation incorporates polar, oxygen-containing functional groups onto hydrophobic polymer backbones, increasing the surface free energy and wettability of the scaffold. These physicochemical modifications influence the behavior of proteins adsorbed onto the material after implantation. For instance, studies showed that increased surface hydrophilicity governs the adsorption of extracellular matrix proteins involved in cell adhesion, such as fibronectin, thereby exposing their binding sites and facilitating integrin-mediated cell attachment, spreading, and proliferation. Furthermore, these surface modifications impact the early innate immune response.

Hydrophobic scaffolds often trigger severe foreign body reactions characterized by pro-inflammatory M1 macrophage polarization and chronic inflammation. In contrast, the mild oxidation by ozone creates a microenvironment that favors an anti-inflammatory, pro-healing M2 macrophage phenotype. In fact, studies by Sunarso et al. have shown that superhydrophilic surfaces generated via ozone gas markedly suppress pro-inflammatory cytokine production in macrophages. Ultimately, this immunomodulatory shift away from chronic inflammation improves the long-term tissue integration and the overall performance of the sterilized scaffold [83].

**Figure 8:**
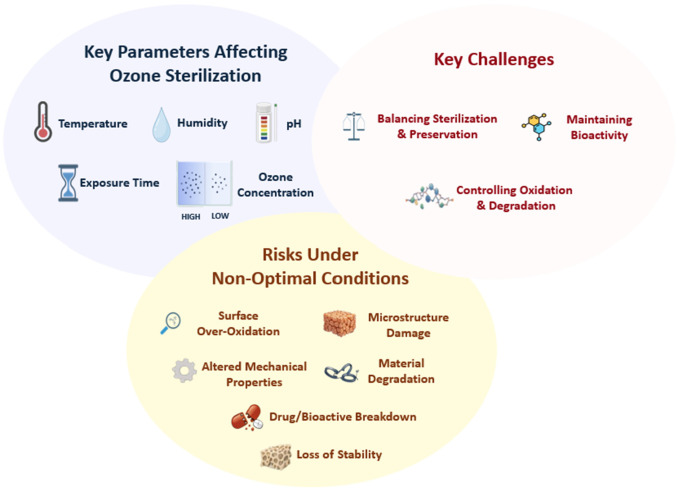
Schematic overview of ozone sterilization in TERM, illustrating the key parameters influencing sterilization efficacy (temperature, humidity, exposure time, pH, ozone concentration), potential risks under non-optimal conditions (over-oxidation, microstructure and mechanical damage, material and bioactive degradation, loss of stability), and the main challenges of the process (balancing sterilization with preservation, controlling oxidation, and maintaining bioactivity).

Despite these biological advantages, the strong oxidative nature of ozone must be carefully controlled to prevent unwanted material damage. The degradation of bioactive molecules or drugs during ozone sterilization, as reported for silicone-based drug delivery hydrogels, can reduce swelling capacity, thermomechanical stability, and lead to drug degradation [1]. Hence, the evaluation of ozone’s effects on mechanical integrity, surface chemistry, and bioactivity is important to determine its suitability as a sterilization method for tissue-engineered scaffolds, as summarized in Figure 8.

### 4.3. Selection Criteria for TERM Scaffold Materials Suitable for Ozone Sterilization

The selection of materials suitable for ozone sterilization in tissue engineering and regenerative medicine scaffolds is a challenging step because of the importance of maintaining the scaffold’s structural, chemical, and biological characteristics. The choice of materials must account for their design, manufacturing process, biocompatibility, and the impact of sterilization on mechanical and biological properties. Because ozone sterilizes via fast surface oxidation, the material’s inherent oxidative sensitivity and its microstructural characteristics are important in determining how well it can be sterilized with this method.

The effect of sterilisation on polymers should be addressed early during scaffold design, as the thickness and density of material can affect sterilant penetration. The gaseous nature of ozone provides better diffusion than liquid sterilants; however, its oxidative reactivity can influence how deeply it penetrates polymers. Its transport within porous biomaterials is governed by coupled diffusion–reaction limitations. Ozone is rapidly consumed at scaffold surfaces through strong oxidative reactions, generating steep concentration gradients that restrict penetration into deeper regions of the construct. As a result, sterilization is predominantly surface-dominated, particularly in high-aspect-ratio or densely structured scaffolds. This may limit effective exposure of microorganisms located within internal pores or poorly interconnected microarchitectures, increasing the risk of incomplete internal sterilization if process parameters are not carefully optimized. Therefore, scaffold architecture and geometry must be considered as key determinants of sterilization efficiency in ozone-based processes. This limitation is particularly relevant for thick or clinically relevant three-dimensional constructs such as bone scaffolds, vascular grafts, and hydrogel-based systems, where internal sterility is essential for safe implantation [11].

The growing use of polymer materials in TERM applications shows the need for understanding the sterilization effects on biocompatibility, chemical stability, and physical integrity. Post-sterilization, the scaffold must remain sterile, non-toxic, and safe. Because no single sterilization technique is universally appropriate for all biomaterials, the choice of method must balance sterilization efficacy with preservation of scaffold architecture [11].

Biodegradable scaffolds have additional challenges in sterilization. Although traditional sterilization methods have long been applied to such materials, they often fail or damage biomaterials. This is problematic for sensitive materials, such as hydrogels, which are difficult to sterilize due to their high water content and vulnerability. Water can begin hydrolytic reactions or alter the polymer network, compromising mechanical strength and functionality. When the application allows it, hydrogels may be dried before sterilization and subsequently rehydrated; however, this approach is unsuitable for many biomedical applications (e.g., bioinks, contact lenses). As a result, it is necessary to take into account the material’s chemical composition, origin, and environmental responsiveness. Despite major advances in hydrogel science, the field of hydrogel sterilization remains comparatively underexplored in the current literature [1].

Importantly, the challenge becomes even greater in cell-laden hydrogels, where encapsulated mammalian cells are substantially more sensitive to oxidative stress than microorganisms. Given the well-established sensitivity of mammalian cells to reactive oxygen species (ROS), ozone exposure may potentially compromise cell viability through oxidative damage mechanisms including lipid peroxidation, mitochondrial dysfunction, and DNA damage. Consequently, while ozone sterilization may be suitable for acellular hydrogel scaffolds prior to cell seeding, direct terminal sterilization of living cell-laden constructs remains insufficiently validated. Future studies should therefore investigate whether carefully optimized ozone exposure conditions, antioxidant protection strategies, or ROS-scavenging hydrogel formulations can preserve cell viability while maintaining sterility assurance [1,84].

The most demanding material selection scenarios arise when the core component of a medical device is difficult to sterilize and critical to performance, while its secondary components (e.g., bioactive molecules, bioresorbable polymers, or electronic elements) are also sensitive. The best strategy is to identify a sterilization method compatible with the main structural material, and then choose secondary materials that can withstand the same sterilized conditions [10].

## 5. Ozone Sterilization Applications in Tissue Engineering and Regenerative Medicine

Ozone sterilization has emerged as a promising alternative to conventional sterilization methods for medical devices and scaffolds used in tissue engineering and regenerative medicine. Unlike ethylene oxide (EtO), steam autoclaving, or gamma irradiation, ozone and UV/ozone treatments provide effective microbial inactivation while avoiding toxic chemical residues and minimizing thermal or radiolytic damage to delicate polymeric structures. Several studies have demonstrated that ozone exposure introduces oxygenated functional groups such as hydroxyl, carbonyl, and carboxyl moieties onto polymer surfaces, thereby increasing surface polarity and hydrophilicity. This physicochemical modification enhances protein adsorption from the extracellular matrix, promotes integrin-mediated adhesion, and facilitates cell spreading and mechanotransduction, which are essential for downstream proliferation and lineage-specific differentiation in engineered tissues [22,23]. Importantly, ozone sterilization preserves scaffold morphology and mechanical integrity, maintaining fiber diameter, porosity, and tensile properties under controlled dosing conditions, whereas steam and radiation often induce chain scission, pore collapse, or dimensional drift [24,25].

From a biological perspective, ozone-treated scaffolds have consistently shown improved cell adhesion and proliferation compared to EtO-treated or autoclaved counterparts. Enhanced wettability and nanoroughness after UV/ozone treatment support focal adhesion formation and cytoskeletal organization, enabling mesenchymal stem cells to undergo osteogenic or chondrogenic differentiation, endothelial cells to form vascular structures, and keratinocytes to re-epithelialize surfaces [14,26,27]. Moreover, ozone decomposes to oxygen and does not leave harmful residues, reducing the risk of inflammatory responses associated with EtO sterilization [2,4]. This residue-free nature makes ozone particularly suitable for scaffolds that are seeded with cells or bioactive molecules shortly after sterilization.

**Figure 9:**
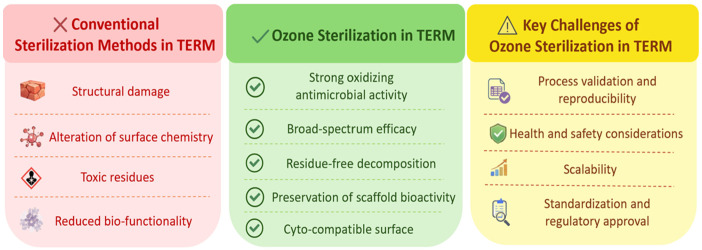
Traditional sterilization methods used in TERM often cause structural and chemical degradation, leave behind toxic residues, and reduce bio-functionality. In contrast, ozone offers strong, residue-free antimicrobial effects; however, reproducibility, safety, scalability, and regulatory approval pose ongoing challenges.

A critical question, however, concerns ozone’s corrosive nature and whether it negatively affects scaffolds containing proteins or bioactive coatings. Indeed, ozone is a strong oxidant, and uncontrolled exposure can degrade sensitive biomolecules. Studies have shown that prolonged or high-concentration ozone treatment can denature proteins or alter peptide-functionalized surfaces, potentially reducing bioactivity [17]. Nevertheless, when ozone sterilization is carefully controlled, using optimized exposure times, concentrations, and humidity, the oxidative effects remain largely confined to the polymer surface and do not significantly damage embedded proteins or compromise scaffold functionality. In fact, mild oxidation can even enhance bioactivity by increasing the availability of binding sites for ECM proteins and growth factors. Thus, the key lies in process optimization: ozone sterilization must be validated for each scaffold type, especially those incorporating protein coatings or drug delivery systems, to balance sterilization efficacy with preservation of biological function, as summarized in Figure 9.

### 5.1. TERM-Specific Applications

Effective ozone sterilization requires high humidity (>80%) to facilitate microbial inactivation. However, this combination of humidity and oxidative reactivity can create compatibility challenges, particularly for polymeric and composite scaffolds. Polymers exhibit the broadest range of responses: their oxidative resistance and structural stability under humid ozone conditions depend on chemical composition and surface hydrophilicity [11].

The literature on ozone sterilization in tissue engineering and regenerative medicine (TERM) highlights several specific applications. In bone tissue engineering, studies such as the University of Bath’s work on electrospun polycaprolactone (PCL) scaffolds show that ozone sterilization effectively eliminates microbial contamination while preserving scaffold morphology and mechanical integrity. Importantly, ozone-treated PCL scaffolds maintained cell compatibility, supporting adhesion and proliferation of osteoblast-like cells, which is critical for bone regeneration [22,85,86,87]. This makes bone scaffolds one of the clearest TERM applications where ozone has been validated. Recent studies further demonstrated that controlled ozone treatment of electrospun PCL scaffolds improves surface wettability through the incorporation of oxygen-containing functional groups while preserving fibrous morphology and mechanical integrity, ultimately enhancing cellular proliferation and scaffold biocompatibility [29].

In cartilage regeneration, medical ozone therapy has been investigated as a treatment modality for osteoarthritis. Reviews emphasize its ability to modulate inflammation, improve oxygenation, and stimulate cartilage repair processes [29]. Although this is more of a therapeutic application than scaffold sterilization, it demonstrates ozone’s bioactivity in regenerative contexts, suggesting synergy when scaffolds are used in cartilage TERM.

For nerve tissue engineering, direct scaffold sterilization studies are limited, but the underlying mechanism—ozone introducing oxygenated groups, increasing hydrophilicity, and enhancing protein adsorption—applies equally to nerve conduits and electrospun nanofiber scaffolds. Improved wettability and ECM protein binding are beneficial for Schwann cell adhesion and axonal guidance, making ozone sterilization theoretically suitable for neural scaffolds. Similarly, in vascular and skin tissue engineering, ozone-treated electrospun scaffolds provide favorable surfaces for endothelial and keratinocyte adhesion, proliferation, and differentiation, supporting angiogenesis and re-epithelialization.

Regarding bioactivity, ozone is most clearly documented in cartilage and bone contexts, where its anti-inflammatory and redox-modulating effects directly support regeneration [29]. In polymeric scaffolds, ozone’s bioactivity is indirect: it enhances surface chemistry to favor cell-material interactions. In these cases, mechanical properties are minimally affected, making electrospun polymer scaffolds (PCL, PU, PLA) the easiest and safest TERM applications for ozone sterilization. By contrast, scaffolds incorporating proteins, peptides, or growth factors are more vulnerable: ozone can oxidize and denature these biomolecules if exposure is not carefully controlled. Thus, while ozone is broadly applicable across TERM, its use is most straightforward and beneficial in synthetic polymer scaffolds where mechanical properties are preserved and bioactivity is enhanced through surface modification.

In conclusion, Ozone sterilization is already validated in bone tissue engineering and cartilage regeneration, and its physicochemical effects make it generalizable to other TERM applications such as nerve, vascular, and skin tissue engineering, as summarized in Table 7. It is most bioactive in cartilage and bone contexts, while being easiest to apply in synthetic polymer scaffolds where mechanical properties are preserved. Protein-coated scaffolds require careful optimization to avoid oxidative damage. Overall, ozone offers a dual advantage: effective sterilization plus surface biofunctionalization, making it a strong candidate across TERM fields [22].

### 5.2. Comparative Analysis with Conventional Sterilization Methods

Terminal sterilization is a critical determinant of both safety and functional performance in medical devices and tissue-engineered constructs. As additive manufacturing and polymer-based scaffolds proliferate, there is an increasing demand for sterilization methods that impose minimal physical and chemical stress, avoid toxic residues, and preserve scaffold architecture and biofunctionality. While conventional sterilization methods remain standard due to their validated efficacy and regulatory recognition, each presents significant limitations for tissue engineering and regenerative medicine (TERM) applications.

EtO’s low-temperature operation and excellent penetration into porous or complex-geometry devices make it valuable for thermally labile materials. However, concerns remain regarding toxic residuals, prolonged aeration requirements, and potential alterations in polymer surface properties that may affect scaffold biocompatibility and subsequent cellular interactions. Validation under ISO 11135 [19] ensures safety and reproducibility but adds procedural complexity and operational costs. Importantly, increasing regulatory pressure due to EtO’s classification as a carcinogen and its environmental burden has led to active phase-out initiatives and removal of EtO sterilization facilities, especially within the EU and parts of the US, further challenging its long-term viability in TERM workflows. As a result, while mature and effective, EtO sterilization is only moderately suitable for TERM when immediate post-sterilization use is required, and its diminishing regulatory acceptance further limits future applicability [4,5,6,8,10,11,93].

Steam autoclaving relies on moist heat to denature microbial proteins and nucleic acids, offering rapid, reliable, and residue-free sterilization. Its high efficacy and well-characterized processes make it ideal for metallic instruments and heat-stable polymers. However, high temperature and humidity are deleterious for most polymeric scaffolds commonly used in TERM, including electrospun fibers, hydrogels, and thermosensitive 3D prints. Exposure often causes hydrolytic degradation, pore collapse, loss of porosity, swelling, and irreversible structural deformations—all incompatible with the precise architecture needed for cell infiltration, nutrient diffusion, or mechanical performance. Consequently, steam sterilization is largely unsuitable for polymeric TERM scaffolds [1,4,11].

Ionizing radiation (gamma or electron-beam) provides deep penetration, a broad biocidal spectrum, and the absence of chemical residues, making it widely adopted in disposable medical device sterilization. However, high-energy radiation generates free radicals, leading to chain scission, crosslinking, or oxidation in polymer backbones. This can result in reduced molecular weight, altered mechanical properties, increased brittleness, discoloration, and changes in surface chemistry. The severity of these effects depends on dose, polymer chemistry, morphology, and load density. Although ISO 11137 [94] provides validated protocols and regulatory acceptance, the high doses required to achieve a sterility assurance level of 10^−6^ often exacerbate material degradation. Thus, for TERM scaffolds where fiber architecture, porosity, or bioactivity are critical, radiation sterilization may compromise performance [8,10,14,16,17].

Vaporized hydrogen peroxide (VHP) sterilization has emerged as a low-temperature alternative, operating via reactive oxygen species generated from hydrogen peroxide vapor. It leaves minimal residues, which are removed by aeration, and short cycle times make it attractive for rapid device turnaround. VHP demonstrates favorable compatibility with many polymers, including 3D-printed scaffolds, and its regulatory maturity has increased following FDA recognition as an “established” sterilization modality in 2024. However, penetration into dense or lumen-containing scaffolds can be limited, and repeated cycles may affect sensitive polymer matrices or coatings. For TERM scaffolds with simple geometry and open porosity, VHP is practical, but for complex or highly porous constructs, empirical validation remains essential [4,10,16,27].

Ozone sterilization combines broad-spectrum antimicrobial activity with the advantage of low-temperature operation and residue-free decomposition to molecular oxygen. Ozone inactivates microorganisms through oxidative attack on membranes, proteins, and nucleic acids, and its gas-phase delivery allows diffusion into porous or fibrous scaffolds. Ozone exposure can also enhance surface properties by introducing hydrophilic functional groups that improve wettability, protein adsorption, and cell adhesion. This mechanism differs fundamentally from that of supercritical CO_2_ (scCO_2_), another emerging low-temperature sterilization technology discussed earlier in this review. Whereas ozone sterilization relies primarily on oxidative reactions and reactive oxygen species, scCO_2_ exerts its effects mainly through diffusional penetration, membrane destabilization, intracellular pH reduction, and polymer plasticization under high-pressure conditions. Consequently, the associated material risks also differ: ozone may induce oxidative degradation or chain scission in oxidation-sensitive polymers, whereas scCO_2_ may alter scaffold morphology or mechanical behavior through transient swelling and pressure-induced structural modifications [1,5,6,11,56]. These mechanistic differences are particularly important in tissue engineering applications, where preservation of scaffold microarchitecture and biofunctionality is critical.

Operationally, ozone sterilizers require an ozone generator, controlled humidity and temperature, and catalytic destruct systems; no consumable sterilant is needed, and capital/operational costs are moderate. However, ozone remains an emerging technology: no harmonized ISO standard exists, and material-specific validation, including extractables/leachables, cytotoxicity, and long-term mechanical testing, is mandatory. Overexposure may induce polymer chain scission, surface oxidation, or discoloration, particularly in elastomers or polymers with vulnerable double bonds. Ozone is thus most suitable for acellular synthetic scaffolds such as PCL, PLGA, or PMMA, where low thermal stress and preservation of scaffold architecture are critical.

Importantly, a substantial portion of the current evidence regarding ozone antimicrobial efficacy and disinfection kinetics originates from environmental applications, including water treatment, wastewater remediation, and air decontamination systems [21,32,33,36,37,38,39,40,41,42]. Although these studies provide valuable mechanistic insight into ozone-mediated microbial inactivation, direct translation to terminal sterilization of implantable tissue-engineered constructs remains challenging. Unlike environmental disinfection systems, TERM scaffolds require simultaneous preservation of mechanical integrity, surface chemistry, porosity, bioactivity, and cytocompatibility while achieving validated sterility assurance levels. In addition, scaffold architecture, diffusion limitations, and the presence of bioactive molecules or living cells may significantly influence ozone penetration and oxidative interactions. Consequently, dedicated biomaterial-specific validation studies remain necessary before conclusions drawn from environmental ozonation systems can be reliably extrapolated to clinical TERM sterilization workflows.

In conclusion, while conventional sterilization methods remain indispensable for many medical devices due to validated performance, regulatory acceptance, and operational maturity, their limitations—especially regarding scaffold integrity, residues, and compatibility with TERM-relevant materials—are substantial. Ozone offers a unique combination of residue-free sterilization, potential surface biofunctionalization, low thermal stress, and gas-phase diffusion that align well with TERM requirements. Its adoption requires rigorous process standardization, material-specific validation, and regulatory harmonization to ensure reproducible sterility and scaffold performance as summarized in Table 8.

## 6. Future Perspectives and Research Gaps

Despite the growing interest in ozone sterilization as a next-generation solution for tissue-engineered constructs, substantial research gaps remain that must be addressed before widespread clinical adoption is possible. Current literature frequently positions ozone as a “promising method” while acknowledging recent developments in ozone-based technologies and equipment, such as microwave UV–ozone generators, that hint at a shift toward more automated and scalable systems [3,5,6,95]. However, the evidence base is still fragmented and far from sufficient to support standardized implementation across the diverse landscape of biomaterials used in TERM.

A primary limitation lies in the need for broader and more systematic experimental studies. Existing investigations into material compatibility, sterilization kinetics, and structure–property relationships often remain restricted to a narrow set of polymers or scaffold types, leaving substantial uncertainty regarding ozone’s performance across newly developed or more complex constructs [3]. Particularly absent are comparative, long-term studies that evaluate how ozone affects the physical, chemical, and mechanical properties of biomaterials with varying compositions, densities, and architectures. The influence of cumulative exposure, repeated sterilization cycles, and storage conditions also remains insufficiently characterized.

Another major research gap concerns the biological response to ozone-sterilized materials. While several studies demonstrate enhanced surface wettability, improved protein adsorption, and favorable cell adhesion profiles following ozone treatment, systematic examinations of cell growth, proliferation, and lineage-specific differentiation on ozone-sterilized nanofibrous mats are still lacking [96]. Such studies are essential for determining whether the oxidative modifications imparted by ozone uniformly support, or potentially hinder, cell-material interactions across scaffold types.

Hydrogels represent one of the most urgent and challenging areas for future investigation. Their high water content, soft mechanical nature, and sensitivity to oxidative stress make sterilization particularly difficult, yet efficient ozone-based protocols for hydrogels remain largely undeveloped. Recent observations highlight that hydrogel sterilization has been comparatively ignored in the literature until very recently, underscoring the necessity for rigorous studies that examine whether ozone can sterilize hydrogels without inducing network collapse, altered swelling behavior, or degradation of encapsulated biomolecules [1]. This gap is particularly important for bioink formulations and injectable hydrogel systems used in bioprinting and minimally invasive therapies.

Standardization remains another fundamental challenge. As a relatively new sterilization approach, ozone lacks harmonized ISO or EN guidelines, resulting in inconsistent protocols and variable outcomes across laboratories. Future work must focus on developing robust, universally accepted sterilization parameters that define ozone concentration, humidity, exposure duration, and validation requirements for different classes of biomaterials. Such standardization is also tightly linked to the development of industrial-scale ozone units capable of delivering uniform treatment across large batches of scaffolds.

An additional research direction involves the examination of combined sterilization factors. Evidence suggests that hybrid strategies—such as ozone-radiation or ozone-plasma combinations—may enhance sterilization efficacy or reduce the dose-dependent oxidative damage observed in sensitive materials. These hybrid methods may be particularly beneficial for dense tissues, bone implants, or composite constructs where single-modality sterilization is insufficient or potentially harmful [17]. Understanding synergies and antagonisms between sterilization modalities will be critical for optimizing protocols that achieve both sterility and material preservation.

Furthermore, despite early demonstrations of ozone integration into biomanufacturing environments, the scalability and automation of ozone processes for large-scale production remain insufficiently addressed in the current literature. While ozone-based systems are evolving toward more practical deployment, detailed insights into supply chain integration, workflow compatibility with GMP manufacturing, and cost–benefit analyses of large-scale ozone sterilization are underdeveloped. This lack of data is especially relevant for 3D printing and automated bioprinting facilities, where sterilization must be seamlessly integrated into continuous, closed-loop production systems [2].

Despite promising results, a deeper understanding of ozone–biomaterial interactions at the molecular and nanoscale level remains essential. Studies in soft matter systems have demonstrated that oxidative environments can induce subtle but critical changes in polymer network organization, interfacial properties, and mechanical behavior, even when bulk properties appear preserved [97]. These findings underscore the need for systematic investigations into long-term stability, especially for multifunctional scaffolds incorporating bioactive molecules, nanostructures, or responsive elements.

Finally, the field must expand toward rigorous biological and preclinical testing. Future investigations should focus on evaluating cell viability, metabolic activity, immunomodulatory responses, and differentiation capacity on ozone-sterilized scaffolds. Equally important will be in vivo studies that assess how ozone-modified surfaces influence inflammation, host integration, degradation kinetics, and long-term tissue remodeling. Such translational research is essential to bridge the gap between promising laboratory findings and reliable, safe clinical applications.

Collectively, these research gaps highlight the considerable work ahead. Addressing them will require coordinated, interdisciplinary efforts spanning materials science, sterilization engineering, cell biology, and regulatory science. Only through such comprehensive and systematic inquiry can ozone sterilization reach its full potential as a safe, effective, and scalable technology for next-generation biomedical manufacturing.

### Limitations and Uncertainties of Ozone Sterilization for TERM

Despite its advantages, ozone sterilization has several limitations that must be explicitly considered before clinical translation. First, penetration into porous scaffolds is not governed solely by gaseous diffusion. Ozone is consumed by reactive surfaces, residual proteins, salts, leachables, and packaging materials, meaning that highly porous but chemically reactive constructs may show strong ozone gradients between the external surface and internal pore network. Therefore, scaffold porosity, pore interconnectivity, tortuosity, water content, and surface chemistry should be treated as process variables rather than passive material features. Worst-case validation should include dense regions, blind pores, multilayered constructs, and hydrated compartments, because these are the areas most likely to receive a lower effective CT dose.

Second, ozone may react with biomaterial-derived leachables, buffer residues, or residual salts to form secondary oxidants or low-molecular-weight by-products. In aqueous systems, ozonation of bromide-containing residues can generate bromate, while reactions with organic matter may yield aldehydes, ketones, carboxylic acids, and peroxides [98]. Although these reactions are well established in water-treatment chemistry, they have not been sufficiently investigated for TERM materials, where residual culture media, chloride- or bromide-containing buffers, plasticizers, unreacted monomers, peptides, lipids, or degradation products may be present. Consequently, ozone-sterilized TERM scaffolds should be evaluated not only for residual ozone, but also for extractables/leachables, cytotoxicity, oxidative by-products, pH/conductivity changes, and long-term stability during storage.

Third, long-term material stability remains insufficiently characterized. Many studies assess morphology, cytocompatibility, or mechanical properties immediately after ozone treatment, but TERM products may be stored for weeks or months before use. Ozone-induced surface oxidation may continue to influence wettability, chain scission, crosslinking density, degradation rate, and protein adsorption over time, particularly in biodegradable polymers, hydrogels, and protein-functionalized scaffolds. Accelerated aging, real-time storage, repeated-cycle exposure, and post-sterilization degradation studies are therefore necessary to distinguish beneficial surface activation from progressive loss of structural integrity.

Finally, scalability remains an unresolved challenge. Laboratory-scale ozone exposure can be relatively uniform, but large TERM devices, multiple-scaffold batches, and packaged constructs require validated gas distribution, humidity control, temperature control, ozone monitoring, and catalytic destruction at the production scale. Integration into GMP biomanufacturing workflows will require closed-chamber automation, reproducible sensor placement, parametric release criteria, and compatibility with aseptic transfer to downstream cell-seeding or packaging steps. These limitations do not negate ozone’s promise, but they define the experimental and regulatory conditions under which ozone can become a reproducible TERM sterilization platform.

## 7. Conclusions

Ozone sterilization is a promising low-temperature, residue-free strategy for TERM materials because it combines broad oxidative antimicrobial activity with the potential to preserve, and in some cases improve, scaffold surface bioactivity. Compared with heat, radiation, and EtO-based sterilization, ozone is particularly attractive for acellular, porous, thermosensitive polymeric constructs such as electrospun scaffolds, PMMA/PCL/PLGA-based matrices, and selected hydrogel systems, provided that concentration, exposure time, humidity, temperature, and material compatibility are rigorously optimized.

At the same time, ozone should not be presented as a universal solution. Its clinical translation depends on quantitative process windows, humidity-controlled sporicidal validation, ozone-specific biological indicators, time-resolved concentration monitoring, assessment of sublethal injury, extractables/leachables testing, and long-term stability studies. Future work should therefore shift from proof-of-concept ozone exposure toward standardized, material-specific, SAL-driven protocols that integrate microbial lethality with preservation of scaffold architecture, bioactivity, and regulatory safety requirements.

## Figures and Tables

**Table 1:** Basic physicochemical properties of ozone [31].

**Parameter**	**Value**
Molecular mass	47.998 g mol^−1^
Density at 273.15 K	2.144 kg m^−3^
Melting point at 1 atm	80.7 K
Boiling point at 1 atm	161.3 K
Critical temperature	261.05 K
Critical pressure	54.62 atm
Critical density	463 kg m^−3^
Density at 90.15 K	1751 kg m^−3^
Viscosity at 90.15 K	1.55 ± 0.02 mPa s^−1^
Electron affinity	1.9–2.7 eV
Dipole moment	0.53 ± 0.02 D
Dielectric constant at 273.15 K	1.0019
Dielectric constant at 90.15 K	4.75
Heat of evaporation	0.71 J mol^−1^
Free energy of formation	6.89 J mol^−1^
Heat of dissolution	0.93 J mol^−1^
Diffusion coefficient in water	1.74 × 10^−9^ m^2^ s^−1^
**Van der Waals’** **C** **onstants**	**Value**
a	3.545 atm L^2^ mol^−2^
b	0.04903 L mol^−1^

**Table 2:** Oxidation-reduction potential of compounds and chemical elements [33].

Chemical Compounds & Elements	Oxidation-Reduction Potential (V)
F^−^	2.87
OH^−^	2.86
S_2_O_8_^2−^	2.60
O^2−^	2.42
O_3_	2.07
H_2_O_2_	1.78
Cl^−^	1.36
ClO_2_	1.27
O_2_	1.23

**Table 3:** Comparative Regulatory and Standardization Characteristics of Class A and Class B Sterilization Methods.

Sterilization Method Categories	Class A	Class B
Sterilization Methods	Gamma Irradiation, E-beam, EtO, Dry Heat, Steam	H_2_O_2_ Gas Plasma, Ozone, Flexible Bag EtO
Standardization	Supported by consensus standards from ISO and other third-party regulatory bodies	No consensus standards Guidance only
FDA Compliance	Considered compliant if standards are followed	Must align with previously FDA-evaluated parameters to avoid classification as “novel”
Documentation for PMA	Lower documentation requirements	Higher documentation requirements necessitate additional validation protocols and thorough descriptions of the sterilization methodology

**Table 5:** Risk Categories of Medical Devices and Processing Requirements [61].

Classification	Patient Contact	Example Devices	Minimum Level of Contamination	Sterility Assurance Level (SAL)	Preferred Methods
Critical	Sterile tissues or vascular system. Devices penetrate the body or contact sterile fluids.	Implants, surgical instruments, endoscopes.	Thorough cleaning and sterilization.	10^−6^	EtO, H_2_O_2_ gas plasma, dry heat.
Semi-Critical	Mucous membranes or non-intact skin. Do not penetrate sterile body areas, but may transmit pathogens.	Endoscopes (GI or respiratory), Laryngoscope blades.	High-level disinfection after cleaning.	≥10^−3^	Glutaraldehyde, peracetic acid.
Non-Critical	Intact skin only. Pose minimal infection risk.	Stethoscopes, blood pressure cuffs.	Low- to intermediate-level disinfection.	10^−3^	Chlorine, alcohols, quaternary ammonium, phenols.

**Table 6:** Interaction of ozone sterilizer with materials used in TERM [1,2,5,6,11,17,56].

Type of Material	Material	Ozone Sterilization Compatibility *	Notes
High-resistance polymer	Polyethylene (PE)	Excellent	Minimal degradation. Low- and medium-density PE grades show more resistance than high-density forms.
	Polypropylene (PP)		Maintains structure and mechanical properties after exposure.
	Fluoropolymers (PTFE, PVDF, ETFE, FEP)		Chemically inert; no change even after prolonged exposure.
	Polycarbonate (PC)		Retains transparency and strength; mild oxidation may improve surface energy.
	Polyether ether ketone (PEEK)		Stable after multiple cycles; ideal for reusable scaffolds.
Moderate-resistance polymer	Polysulfone (PSF)	Good	Slight surface modification and decreased gloss.
	Polyhydroxybutyrate (PHB)		Increased modulus and elongation post-sterilization.
	Polyamide (Nylon)		Partial chain cleavage, Risk of depolymerization and brittleness.
	Poly(methyl methacrylate) (PMMA)		Minor surface changes after 10–100 cycles.
	Silicone hydrogel		Effective at low doses; higher doses increase ionic permeability, alter surface roughness, and reduce mechanical stability.
	Chitosan hydrogel nanoparticles		Retains structure and chemical composition after sterilization; may exhibit mild cytotoxicity and lower efficacy compared with gamma irradiation.
Other polymers	Polystyrene (PS)	Fair	Surface damage after <3 cycles; minor ozone adsorption effects.
	Cellulosics		Partial oxidation and loss of mechanical strength; cellulose acetate more stable.
Low-resistance polymer	Polyurethane (PU)	Poor	Rapid oxidation and loss of elasticity due to unsaturated bonds.
Metal	Stainless steel (304, 316), titanium	Excellent	No morphological changes; surface oxidation may improve chemical reactivity and osteointegration.
Ceramic	Zirconia, hydroxyapatite	Excellent	No detectable structural or chemical degradation.
Composite	Polymer composites	Variable	Ozone can induce morphological and mechanical alterations; optimization is required.

* Excellent: These materials are unaffected by ozone and can retain their integrity indefinitely. Good: Ozone has a minimal impact on these materials, but continuous exposure to high ozone levels will cause breakdown or corrosion. Fair: These materials begin to degrade within weeks of ozone exposure, and prolonged use at any concentration leads to breakdown or corrosion. Poor: Ozone damages these materials within hours or days, making them unsuitable for any ozone application. Variable: The ozone resistance of polymer composites depends on factors like the polymer matrix, filler composition, and interfacial stability (Ozone Compatible Materials).

**Table 7:** Comparative evidence across included works in case of use of Ozone in TERM [2,4,14,17,22,23,24,25,26,27,29,88,89,90,91,92].

Theme	What the Evidence Shows	Why It Matters for TERM
Ozone as an effective sterilant	Ozone/UV-ozone effectively reduces bioburden while avoiding toxic residuals typical of EO; it imposes low thermal stress versus steam and preserves polymer integrity with appropriate dosing.	Sterile, residue-free, and structurally intact scaffolds reduce early inflammatory risk and are immediately cell-compatible for TE workflows.
Surface chemistry and wettability tuning	Ozone introduces oxygenated functional groups (–OH, –C=O, –COOH), increases surface energy, and lowers contact angle; electrospun fiber surfaces exhibit controlled oxidation without core damage.	Enhanced ECM protein adsorption and integrin engagement improve adhesion/spreading and downstream differentiation on scaffolds.
Morphology and porosity retention	SEM/AFM show nanoroughness/topography adjustments rather than collapse; pore networks/interconnectivity remain stable with UV/ozone.	Mass transport (nutrients/O_2_) and cell infiltration remain viable—crucial for thick constructs and vascularization.
Mechanical properties under control	Tensile/compressive properties of electrospun PU/PCL scaffolds are preserved under controlled UV/ozone; EO and steam can degrade or plasticize certain polymers	Load-bearing suitability and dimensional stability are maintained for soft-tissue constructs and early in vivo handling.
Biological outcomes on cells	Improved protein adsorption supports focal adhesion (vinculin/FAK), mechanotransduction (YAP/TAZ, Wnt/β-catenin) and cell functions (adhesion, proliferation, lineage differentiation).	Validates scaffold readiness for TE assays and translation, across bone/cartilage/skin/vascular contexts.
Ozone bioactivity in RM	Medical ozone can modulate local redox/immune signaling and support early tissue repair and angiogenesis when clinically co-applied.	Provides a dual-action framework—sterile scaffold plus microenvironmental support for regeneration.

**Table 8:** Comprehensive Comparison of Sterilization Modalities for TERM-Relevant Materials [1,2,4,5,6,8,10,11,14,16,17,22,23,27,90,93].

Parameter	Ozone (O_3_)	Ethylene Oxide (EtO)	Vaporized Hydrogen Peroxide (VHP)	Steam (Autoclave)	Gamma/E-beam
Typical temperature	Low (25–40 °C)	Low (30–60 °C)	Low (25–50 °C)	High (121–134 °C)	Ambient
Mechanism	Oxidation (ROS)	Alkylation	Oxidation (H_2_O_2_)	Moist heat denaturation	Ionizing radiation (DNA scission)
Residues	Decomposes to O_2_; material-derived oxidation products possible.	Persistent toxic residues; long aeration required.	Minimal; removed by aeration.	None	None
Penetration	Moderate; limited in dense loads or lumen-containing scaffolds.	Excellent; penetrates complex geometries.	Surface-dominant; some penetration with cycle design.	High	Excellent
Sterility assurance level (SAL)	≤10^−6^ depending on concentration, humidity, exposure time (emerging validation)	10^−6^ (validated)	10^−6^ (validated)	10^−6^ (validated)	10^−6^ (validated)
Material compatibility	Variable; good for thermoplastics (PCL, PLGA, PMMA) and electrospun scaffolds; risky for elastomers/unsaturated polymers.	Broad, but residues and long cycles may affect polymers.	Good for many polymers.	Poor for heat-sensitive or hydrolytically labile materials.	Alters polymer chemistry; may affect mechanical properties and degradation.
Cycle time/throughput	2–20 ppm for 4–60 min (laboratory decontamination); 3000–15,000 ppm at 80–90% RH and 25–35 °C in BI-based sterilization studies	37–63 °C, 40–80% RH, 450–1200 mg/L for 1–6 h exposure, followed by prolonged aeration	25–65 °C; cycle duration depends on system configuration and load	Typically 121 °C for 15–20 min or 134 °C short-cycle sterilization	Typical sterilization dose 15–35 kGy; 25 kGy commonly used for medical devices
Regulatory maturity	Emerging; lacks harmonized ISO standard.	High	Increasingly established; FDA recognition 2024	High	High
Operational/cost considerations	Moderate capital cost; on-demand sterilant; programmable cycles; requires ozone generator and abatement system.	Moderate to high; long cycles; strict handling required.	Moderate; requires vapor generator and chamber; short cycles	Low to moderate; standard autoclaves widely available.	High infrastructure and validation cost; dose mapping required.
Effect on materials	Preserves fiber architecture; increases hydrophilicity/functional groups, improving cell adhesion; preserves polymer morphology/mechanics (low thermal stress); risk of chain scission or discoloration at high doses.	Potential plasticization or surface chemistry alteration.	Generally preserves polymer integrity; may be limited in dense/lumen loads.	Hydrolytic degradation (pore collapse risk); dimensional drift (affects electrospun structures).	Chain scission/crosslinking Discoloration (can signal microstructural damage); changes in mechanical properties.
Suitability for TERM scaffolds	High—best balance of sterilization, surface biofunctionality, and mechanical preservation for polymeric scaffolds.	Moderate—effective sterilization but residue management and potential material effects complicate TE cell-seeding timelines.	High for polymer devices; validated cycles available; suitable for simple geometries.	Low for polymeric scaffolds—mechanical/morphological degradation risk is significant.	Moderate—excellent sterilization but risk of mechanical/surface property changes that impair cell interactions.
Additional TERM-specific considerations	Ozone demand may generate concentration gradients in dense, hydrated, or packaged constructs; CT/RH validation is required within worst-case pore architectures	Long aeration phases may delay downstream TERM manufacturing and rapid cell-seeding workflows	Penetration efficiency decreases in long lumens, dense porous scaffolds, and vacuum-sensitive hydrogels; ROS may oxidize sensitive coatings or biomolecules	Heat and moisture may induce swelling, denaturation, and pore collapse in proteins, hydrogels, and bioinks	Radiation-induced free radicals may continue post-sterilization degradation reactions during storage of biodegradable constructs

## Data Availability

No new data were created or analyzed in this study. Data sharing is not applicable to this article.

## Abbreviations

The following abbreviations are used in this manuscript:

CEN	European Committee for Standardization
CFTR	Cystic Fibrosis Transmembrane Conductance Regulator
EtO	Ethylene Oxide
FDA	Food And Drug Administration
GI	Gastrointestinal
IDLH	Immediately Dangerous to Life or Health
IQ	Installation Qualification
MPQ	Microbiological Performance Qualification
NIOSH	National Institute for Occupational Safety and Health
OP	Operational Qualification
PEL	Permissible Exposure Limit
PPE	Personal Protective Equipment
PQ	Performance Qualification
RM	Regenerative Medicine
ROS	Reactive Oxygen Species
SAL	Sterility Assurance Level
STEL	Short-Term Exposure Limit
TE	Tissue Engineering
TERM	Tissue Engineering and Regenerative Medicine
VHP	Vaporized Hydrogen Peroxide
